# Genetic determinants of childhood blood pressure and heart rate in relation to adult health outcomes: the consortium of childhood blood pressure

**DOI:** 10.1093/eurheartj/ehag313

**Published:** 2026-04-27

**Authors:** Tian Xie, Alireza Ani, Ahmad Vaez, Ilja M Nolte, Shaoyong Su, Mami Ishikuro, Siqi Wang, Wenbo Zhang, Ana Goncalves Soares, Ehsan Motazedi, Lucinda Calas, Justiina Ronkainen, Casper-Emil T Pedersen, Xueling Lu, Sara E Stinson, Janine F Felix, Evelina Stankevic, Carol A Wang, Elisabeth Thiering, Dietmar Fernández, Beatriz Calvo-Serra, Maria G Stathopoulou, Ruby Fore, Ashish Kumar, Johanna Tuhkanen, Jose Ramon Bilbao, Jesus Ibarluzea, Aaron Isaacs, Cilius Esmann Fonvig, Fernando Rivadeneira, Morten Asp Vonsild Lund, Louise Aas Holm, Peter J van der Most, Harriëtte Riese, Akira Narita, Gen Tamiya, Claudia Flexeder, Rikstje Wiersma, Anna D Argoty-Pantoja, Rebecca Vinding, Tine Willum Hansen, Thomas Kümler, Marisa Estarlich, Mariona Bustamante, Wen Lun Yuan, Anne Boland-Augé, Jean-François Deleuze, Alexandros M Petrelis, Sheryl Rifas-Shiman, Eero Kajantie, Nora Fernandez-Jimenez, Loreto Santa-Marina, Ilja C W Arts, Jari Lahti, Timo Strandberg, Inger Kull, Anna Bergström, Marie-France Hivert, Klaus Bønnelykke, Shinichi Kuriyama, Lawrence J Beilin, Trevor A Mori, Catharina A Hartman, Niels Grarup, Carel Thijs, Katri Räikkönen, Erik Melén, Emily Oken, Sophie Visvikis-siest, Kristine B Gützkow, Regina Grazuleviciene, Barbara Heude, Leda Chatzi, Martine Vrijheid, Marie Standl, Tanja Vrijkotte, Craig E Pennell, Albertine J Oldehinkel, Torben Hansen, Vincent W V Jaddoe, Jens-Christian Holm, Sylvain Sebert, Nicholas J Timpson, Taku Obara, Xiaoling Wang, Deborah A Lawlor, Eva Corpeleijn, Tarunveer S Ahluwalia, Harold Snieder

**Affiliations:** Guangzhou National Laboratory, No. 9 XingDaoHuanBei Road, Guangzhou International Bio Island, Guangzhou 510005, Guangdong Province, China; Department of Epidemiology, University of Groningen, University Medical Center Groningen, PO Box 30.001, 9700 RB Groningen, The Netherlands; Department of Epidemiology, University of Groningen, University Medical Center Groningen, PO Box 30.001, 9700 RB Groningen, The Netherlands; Department of Bioinformatics, Isfahan University of Medical Sciences, Isfahan, Iran; Department of Epidemiology, University of Groningen, University Medical Center Groningen, PO Box 30.001, 9700 RB Groningen, The Netherlands; Department of Bioinformatics, Isfahan University of Medical Sciences, Isfahan, Iran; Department of Epidemiology, University of Groningen, University Medical Center Groningen, PO Box 30.001, 9700 RB Groningen, The Netherlands; Georgia Prevention Institute, Medical College of Georgia, Augusta University, Augusta, GA, USA; Department of Preventive Medicine and Epidemiology, Tohoku Medical Megabank Organization, Tohoku University, Sendai, Japan; Department of Epidemiology, University of Groningen, University Medical Center Groningen, PO Box 30.001, 9700 RB Groningen, The Netherlands; Department of Epidemiology, University of Groningen, University Medical Center Groningen, PO Box 30.001, 9700 RB Groningen, The Netherlands; MRC Integrative Epidemiology Unit, University of Bristol, Bristol, UK; Population Health Sciences, Bristol Medical School, University of Bristol, Bristol, UK; Department of Public and Occupational Health (POH), Amsterdam UMC, Location University of Amsterdam, Amsterdam, The Netherlands; Amsterdam Public Health Research Institute, Amsterdam, The Netherlands; Inserm, INRAE, Center for Research in Epidemiology and StatisticS (CRESS), Université Paris Cité and Université Sorbonne Paris Nord, Paris, France; Research Unit of Population Health, University of Oulu, Oulu, Finland; COPSAC, Copenhagen Prospective Studies on Asthma in Childhood, Herlev and Gentofte Hospital, University of Copenhagen, Ledreborg Alle 34, 2820 Gentofte, Copenhagen, Denmark; Department of Epidemiology, University of Groningen, University Medical Center Groningen, PO Box 30.001, 9700 RB Groningen, The Netherlands; Laboratory of Environmental Medicine and Developmental Toxicology, Shantou University Medical College, Guangdong, China; Novo Nordisk Foundation Center for Basic Metabolic Research, Faculty of Health and Medical Sciences, University of Copenhagen, Copenhagen, Denmark; The Generation R Study Group, Erasmus MC, University Medical Center Rotterdam, Rotterdam, the Netherlands; Department of Pediatrics, Erasmus MC, University Medical Center Rotterdam, Rotterdam, the Netherlands; Novo Nordisk Foundation Center for Basic Metabolic Research, Faculty of Health and Medical Sciences, University of Copenhagen, Copenhagen, Denmark; School of Medicine and Public Health, University of Newcastle, Newcastle, New South Wales, Australia; Mothers and Babies Research Program, Hunter Medical Research Institute, Newcastle, New South Wales, Australia; Institute of Epidemiology, Helmholtz Zentrum München- German Research Center for Environmental Health, Neuherberg, Germany; Division of Metabolic and Nutritional Medicine, Dr. von Hauner Children’s Hospital, University of Munich Medical Center, Munich, Germany; Cerba Internacional, Barcelona, Spain; ISGlobal, Barcelona, Spain; ISGlobal, Barcelona, Spain; Department of Medicine and Life Sciences, Universitat Pompeu Fabra (UPF), Barcelona 08003, Spain; IGE-PCV, Université de Lorraine, Nancy, France; INSERM U1065, C3M, Université Côte D’Azur, Nice, France; Department of Population Medicine, Harvard Medical School, Harvard Pilgrim Health Care Institute, Boston, MA, USA; Department of Clinical Sciences and Education, Södersjukhuset (SÖS), Karolinska Institutet, Stockholm, Sweden; Department of Psychology and Logopedics, Faculty of Medicine, University of Helsinki, Helsinki, Finland; Department of Genetics, Physical Anthropology and Animal Physiology, University of the Basque Country (UPV/EHU), Leioa, Spain; Biobizkaia Health Research Institute, Immunogenetics Research Laboratory, Barakaldo, Spain; Spanish Biomedical Research Center in Diabetes and Associated Metabolic Disorders (CIBERDEM), Madrid, Spain; Ministry of Health of the Basque Government, Sub-Directorate for Public Health and Addictions of Gipuzkoa, San Sebastián, Spain; Spanish Consortium for Research in Epidemiology and Public Health (CIBERESP), Madrid, Spain; Biogipuzkoa Health Research Institute, Group of Environmental Epidemiology and Child Development, Donostia- San Sebastián, Spain; Department of Physiology, Maastricht University, Postbus 616, Maastricht 6200 MD, the Netherlands; CARIM School for Cardiovascular Diseases, Maastricht University, Postbus 616, Maastricht 6200 MD, the Netherlands; Maastricht Centre for Systems Biology (MaCSBio), Maastricht University, Postbus 616, Maastricht 6200 MD, the Netherlands; Novo Nordisk Foundation Center for Basic Metabolic Research, Faculty of Health and Medical Sciences, University of Copenhagen, Copenhagen, Denmark; The Children’s Obesity Clinic, Accredited European Centre for Obesity Management, Department of Pediatrics, Copenhagen University Hospital Holbæk, Holbæk, Denmark; Department of Clinical Medicine, Faculty of Health and Medical Sciences, University of Copenhagen, Copenhagen, Denmark; The Generation R Study Group, Erasmus MC, University Medical Center Rotterdam, Rotterdam, the Netherlands; Department of Internal Medicine, Erasmus MC, University Medical Center Rotterdam, Rotterdam, the Netherlands; The Children’s Obesity Clinic, Accredited European Centre for Obesity Management, Department of Pediatrics, Copenhagen University Hospital Holbæk, Holbæk, Denmark; Department of Biomedical Sciences, Faculty of Health and Medical Sciences, University of Copenhagen, Copenhagen, Denmark; Novo Nordisk Foundation Center for Basic Metabolic Research, Faculty of Health and Medical Sciences, University of Copenhagen, Copenhagen, Denmark; The Children’s Obesity Clinic, Accredited European Centre for Obesity Management, Department of Pediatrics, Copenhagen University Hospital Holbæk, Holbæk, Denmark; Department of Epidemiology, University of Groningen, University Medical Center Groningen, PO Box 30.001, 9700 RB Groningen, The Netherlands; Department of Psychiatry, University of Groningen, University Medical Center Groningen, Groningen, The Netherlands; Department of Integrative Genomics, Tohoku Medical Megabank Organization, Tohoku University, Sendai, Japan; Department of Integrative Genomics, Tohoku Medical Megabank Organization, Tohoku University, Sendai, Japan; Department of AI and Innovative Medicine, Graduate School of Medicine, Tohoku University, Sendai, Japan; Statistical Genetics Team, Center for Advanced Intelligence Project, RIKEN, Tokyo, Japan; Institute of Epidemiology, Helmholtz Zentrum München- German Research Center for Environmental Health, Neuherberg, Germany; Institute and Clinic for Occupational, Social and Environmental Medicine, University Hospital, LMU Munich, Munich, Germany; Department of Epidemiology, University of Groningen, University Medical Center Groningen, PO Box 30.001, 9700 RB Groningen, The Netherlands; Department of Epidemiology, University of Groningen, University Medical Center Groningen, PO Box 30.001, 9700 RB Groningen, The Netherlands; School of Medicine, National Autonomous University of Mexico (UNAM), Mexico City, Mexico; COPSAC, Copenhagen Prospective Studies on Asthma in Childhood, Herlev and Gentofte Hospital, University of Copenhagen, Ledreborg Alle 34, 2820 Gentofte, Copenhagen, Denmark; Steno Diabetes Center Copenhagen, Borgmester Ib Juuls Vej 83, 2730 Herlev, Denmark; University of Copenhagen, Copenhagen, Denmark; Steno Diabetes Center Copenhagen, Borgmester Ib Juuls Vej 83, 2730 Herlev, Denmark; Spanish Consortium for Research in Epidemiology and Public Health (CIBERESP), Madrid, Spain; Department of Nursing, University of Valencia, Valencia, Spain; Epidemiology and Environmental Health Joint Research Unit, the Foundation for the Promotion of Health and Biomedical Research of Valencia Region, Universitat Jaume I-Universitat de València, Valencia, Spain; ISGlobal, Barcelona, Spain; Spanish Consortium for Research in Epidemiology and Public Health (CIBERESP), Madrid, Spain; Universitat Pompeu Fabra (UPF), Barcelona, Spain; Inserm, INRAE, Center for Research in Epidemiology and StatisticS (CRESS), Université Paris Cité and Université Sorbonne Paris Nord, Paris, France; CEA, Centre National de Recherche en Génomique Humaine (CNRGH), Université Paris-Saclay, Evry, France; CEA, Centre National de Recherche en Génomique Humaine (CNRGH), Université Paris-Saclay, Evry, France; IGE-PCV, Université de Lorraine, Nancy, France; Department of Population Medicine, Harvard Medical School, Harvard Pilgrim Health Care Institute, Boston, MA, USA; Population Health Unit, Finnish Institute for Health and Welfare, Helsinki and Oulu, Finland; Clinical Medicine Research Unit, MRC Oulu, Oulu University Hospital and University of Oulu, Oulu, Finland; Department of Clinical and Molecular Medicine, Norwegian University of Science and Technology, Trondheim, Norway; Children’s Hospital, Helsinki University Hospital and University of Helsinki, Helsinki, Finland; Department of Genetics, Physical Anthropology and Animal Physiology, University of the Basque Country (UPV/EHU), Leioa, Spain; Biobizkaia Health Research Institute, Immunogenetics Research Laboratory, Barakaldo, Spain; Ministry of Health of the Basque Government, Sub-Directorate for Public Health and Addictions of Gipuzkoa, San Sebastián, Spain; Spanish Consortium for Research in Epidemiology and Public Health (CIBERESP), Madrid, Spain; Biogipuzkoa Health Research Institute, Group of Environmental Epidemiology and Child Development, Donostia- San Sebastián, Spain; Maastricht Centre for Systems Biology (MaCSBio), Maastricht University, PO Box 616, Maastricht, the Netherlands; Department of Psychology and Logopedics, Faculty of Medicine, University of Helsinki, Helsinki, Finland; Folkhälsan Research Institute, Helsinki, Finland; Research Unit of Population Health, University of Oulu, Oulu, Finland; University of Helsinki and Helsinki University Hospital, Helsinki, Finland; Department of Clinical Sciences and Education, Södersjukhuset (SÖS), Karolinska Institutet, Stockholm, Sweden; Sachs’ Children’s and Youth Hospital, Södersjukhuset, Stockholm, Sweden; Institute of Environmental Medicine, Karolinska Institutet, Stockholm, Sweden; Centre for Occupational and Environmental Medicine, Region Stockholm, Stockholm, Sweden; Department of Population Medicine, Harvard Pilgrim Health Care Institute, Boston, MA, USA; Diabetes Unit, Department of Medicine, Massachusetts General Hospital, Boston, MA, USA; Harvard Medical School, Boston, MA, USA; COPSAC, Copenhagen Prospective Studies on Asthma in Childhood, Herlev and Gentofte Hospital, University of Copenhagen, Ledreborg Alle 34, 2820 Gentofte, Copenhagen, Denmark; Department of Preventive Medicine and Epidemiology, Tohoku Medical Megabank Organization, Tohoku University, Sendai, Japan; Medical School, Royal Perth Hospital Unit, University of Western Australia, Perth, Western Australia, Australia; Medical School, Royal Perth Hospital Unit, University of Western Australia, Perth, Western Australia, Australia; Department of Psychiatry, University of Groningen, University Medical Center Groningen, Groningen, The Netherlands; Novo Nordisk Foundation Center for Basic Metabolic Research, Faculty of Health and Medical Sciences, University of Copenhagen, Copenhagen, Denmark; Care and Public Health Research Institute, Dept of Epidemiology, Maastricht University Medical Centre+, PO Box 616, Maastricht, The Netherlands; Department of Psychology and Logopedics, Faculty of Medicine, University of Helsinki, Helsinki, Finland; Department of Clinical Sciences and Education, Södersjukhuset (SÖS), Karolinska Institutet, Stockholm, Sweden; Sachs’ Children’s and Youth Hospital, Södersjukhuset, Stockholm, Sweden; Department of Population Medicine, Harvard Pilgrim Health Care Institute, Boston, MA, USA; Harvard Medical School, Boston, MA, USA; IGE-PCV, Université de Lorraine, Nancy, France; Division of Climate and Environmental Health, Department of Air Quality and Noise, Norwegian Institute of Public Health (NIPH), P.O.Box, Oslo, Norway; Department of Environmental Science, Vytautas Magnus University, Kaunas, Lithuania; Inserm, INRAE, Center for Research in Epidemiology and StatisticS (CRESS), Université Paris Cité and Université Sorbonne Paris Nord, Paris, France; Department of Preventive Medicine, University of Southern California, Los Angeles, CA, USA; ISGlobal, Barcelona, Spain; Spanish Consortium for Research in Epidemiology and Public Health (CIBERESP), Madrid, Spain; Universitat Pompeu Fabra (UPF), Barcelona, Spain; Institute of Epidemiology, Helmholtz Zentrum München- German Research Center for Environmental Health, Neuherberg, Germany; German Center for Child and Adolescent Health (DZKJ), partner Site München, Munich, Germany; Department of Public and Occupational Health (POH), Amsterdam UMC, Location University of Amsterdam, Amsterdam, The Netherlands; Amsterdam Public Health Research Institute, Amsterdam, The Netherlands; Amsterdam Reproduction & Development Research Institute, Amsterdam University Medical Centers, Amsterdam, The Netherlands; School of Medicine and Public Health, University of Newcastle, Newcastle, New South Wales, Australia; Mothers and Babies Research Program, Hunter Medical Research Institute, Newcastle, New South Wales, Australia; Maternity and Gynaecology, John Hunter Hospital, Newcastle, New South Wales, Australia; Department of Psychiatry, University of Groningen, University Medical Center Groningen, Groningen, The Netherlands; Novo Nordisk Foundation Center for Basic Metabolic Research, Faculty of Health and Medical Sciences, University of Copenhagen, Copenhagen, Denmark; Faculty of Health Sciences, University of Southern Denmark, Odense, Denmark; The Generation R Study Group, Erasmus MC, University Medical Center Rotterdam, Rotterdam, the Netherlands; Department of Pediatrics, Erasmus MC, University Medical Center Rotterdam, Rotterdam, the Netherlands; Novo Nordisk Foundation Center for Basic Metabolic Research, Faculty of Health and Medical Sciences, University of Copenhagen, Copenhagen, Denmark; The Children’s Obesity Clinic, Accredited European Centre for Obesity Management, Department of Pediatrics, Copenhagen University Hospital Holbæk, Holbæk, Denmark; Department of Clinical Medicine, Faculty of Health and Medical Sciences, University of Copenhagen, Copenhagen, Denmark; Research Unit of Population Health, University of Oulu, Oulu, Finland; MRC Integrative Epidemiology Unit, University of Bristol, Bristol, UK; Population Health Sciences, Bristol Medical School, University of Bristol, Bristol, UK; Department of Preventive Medicine and Epidemiology, Tohoku Medical Megabank Organization, Tohoku University, Sendai, Japan; Georgia Prevention Institute, Medical College of Georgia, Augusta University, Augusta, GA, USA; MRC Integrative Epidemiology Unit, University of Bristol, Bristol, UK; Population Health Sciences, Bristol Medical School, University of Bristol, Bristol, UK; Department of Epidemiology, University of Groningen, University Medical Center Groningen, PO Box 30.001, 9700 RB Groningen, The Netherlands; COPSAC, Copenhagen Prospective Studies on Asthma in Childhood, Herlev and Gentofte Hospital, University of Copenhagen, Ledreborg Alle 34, 2820 Gentofte, Copenhagen, Denmark; Steno Diabetes Center Copenhagen, Borgmester Ib Juuls Vej 83, 2730 Herlev, Denmark; The Bioinformatics Center, Department of Biology, University of Copenhagen, Ole Maaløes Vej 5, 2200 Copenhagen, Denmark; Department of Science and Environment, Roskilde University, Universitetsvej 1, 4000 Roskilde, Denmark; Department of Epidemiology, University of Groningen, University Medical Center Groningen, PO Box 30.001, 9700 RB Groningen, The Netherlands

**Keywords:** Childhood blood pressure, Childhood heart rate, Genome-wide association study, Adult health outcomes

## Abstract

**Background and Aims:**

To elucidate the genetic architecture of blood pressure (BP) and heart rate (HR) during early life and assess their potential relevance to adult health outcomes.

**Methods:**

The largest genome-wide association study (GWAS) meta-analyses to date of childhood systolic BP, diastolic BP, pulse pressure, and mean arterial pressure (*n* = 28 425) and HR (*n* = 22 565) were conducted in children of European ancestry aged 4–17 years. Follow-up analyses included comparisons with adult GWAS results, polygenic risk score (PRS) analyses in independent cohorts of diverse ancestries, and a phenome-wide association study in the UK Biobank.

**Results:**

Eight genome-wide significant loci were identified for childhood BP (*KIAA2013, CACNB2, PLCE1, PAX2, COL4A2, RP11-236L14.1, CFDP1, TPX2*) and three loci for childhood HR (*CCDC141, ACHE, MYH6*); all novel in children but previously reported in adults. Childhood PRSs explained up to 1.6% of BP variance and 5.2% of HR variance among children of European ancestry. Genetic correlations between childhood and adulthood BP traits were moderate (r_g_ = 0.4–0.7), suggesting age-specific genetic effects on BP. In the UK Biobank, higher childhood BP PRS levels were significantly associated with a broad range of adult health outcomes, particularly cardiometabolic outcomes such as hypertension, angina, myocardial infarction, and cardiovascular disease-related mortality.

**Conclusions:**

These findings advance the understanding of the genetic architecture of childhood BP and HR and provide compelling genetic evidence linking childhood BP to a broad spectrum of adult health outcomes—particularly cardiometabolic conditions—which may inform targeted prevention strategies from a young age.

## Graphical Abstract

**Graphical Abstract:**
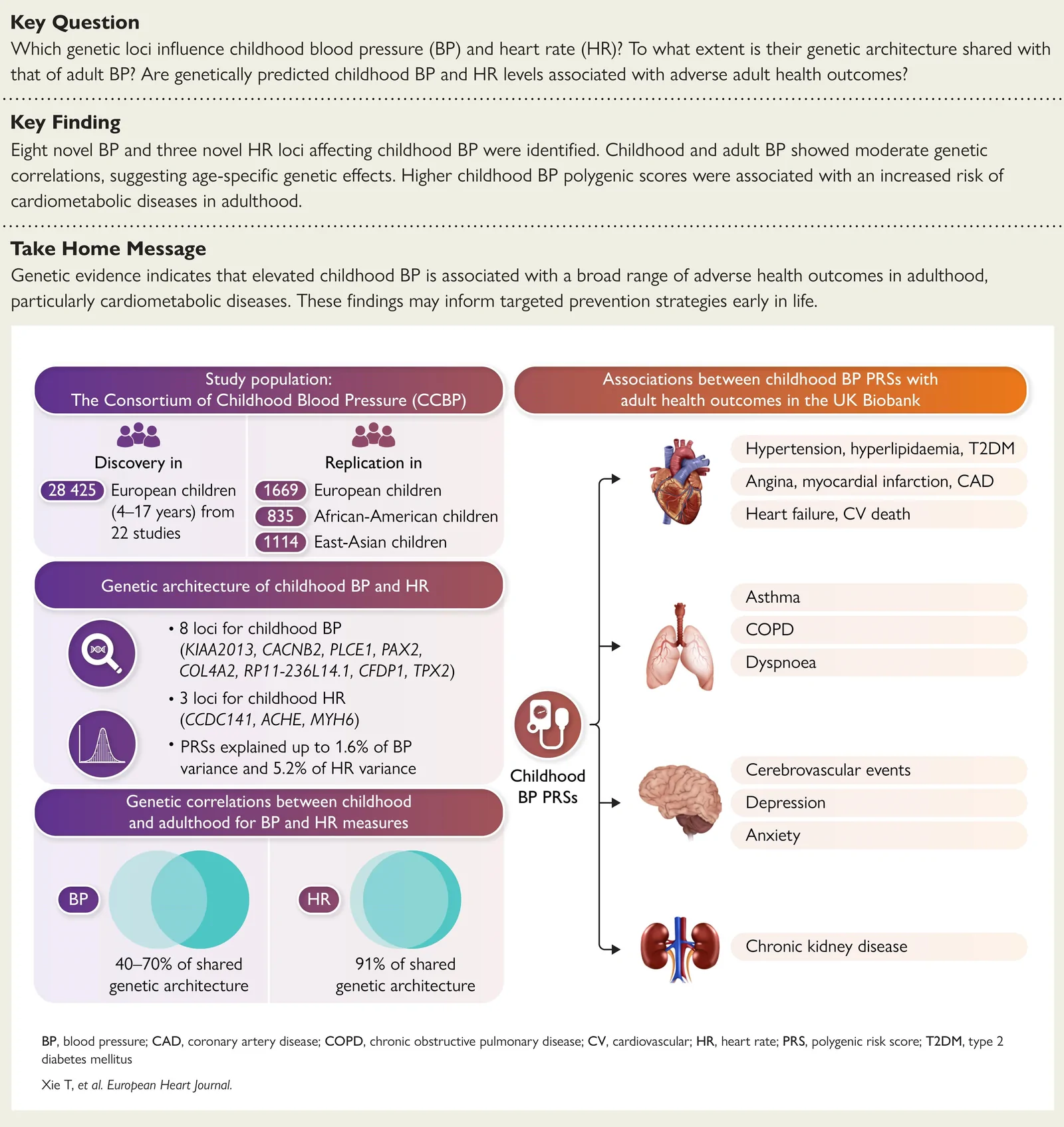
Genetics of childhood BP and HR and relation to adult health.

**See the editorial comment for this article ‘Differences and similarities of the genetic architectures of blood pressure and heart rate in children and adults’, by K. Uddin and H.R. Warren, https://doi.org/10.1093/eurheartj/ehag449.**

## Introduction

High blood pressure (BP) is a major risk factor for coronary artery disease, stroke, and chronic kidney disease worldwide.^1^ It is well-established that 30–60% of the variance in BP can be attributed to genetics,^2–5^ and genome-wide association studies (GWASs) in adults have identified over 2000 BP-related genetic variants.^6,7^ Similarly, resting heart rate (HR) is a heritable trait^8–10^ linked to a range of cardiovascular outcomes,^11^ including overall mortality.^12,13^ Recent GWASs have reported more than 350 loci associated with HR in adults.^14,15^

Despite these advances in adults, the genetic architecture of BP and HR during childhood remains poorly understood. This gap is particularly important because longitudinal studies have shown that elevated BP in childhood and adolescence is associated with an increased risk of adult hypertension and cardiovascular disease,^16,17^ suggesting that the origins of cardiovascular diseases may lie in early life. Moreover, previous studies indicated that genetic effects on BP might vary with age.^18–20^ Different genetic variants may play a role in BP regulation in childhood, or the effect of the same variants might also change over time. Identifying genetic risk factors in childhood could therefore improve our understanding of the early origins of hypertension and cardiovascular disease, facilitate early risk stratification, and clarify the contribution of genetic variation independent of antihypertensive medication use, which is rare in paediatric populations. More broadly, identifying genetic variants for early-life exposures such as BP and HR is essential for elucidating life course effects to support the timing of effective preventive interventions.^21^

However, genetic studies of BP and HR in children have lagged behind those in adults, largely due to smaller sample sizes and the challenges of recruiting children. To date, only two GWASs of childhood BP in predominantly European ancestry has been published. One study identified two genome-wide significant systolic BP (SBP) loci (one specific to prepuberty and one to puberty)^22^ and the other reported no genome-wide significant loci,^23^ with the authors acknowledging the limited sample size and the need for larger GWAS consortia on childhood BP. To our knowledge, no GWAS has yet explored the genetic basis of resting HR in children. Moreover, none of these studies investigated the associations between genetic risk for childhood BP or HR and adult health outcomes, leaving an important gap in understanding the long-term health implications of early-life genetic risk.

This study represents the first report from the Consortium of Childhood Blood Pressure (CCBP), established to fill these gaps by enabling adequately powered genetic studies of BP and HR in children. To address these questions, the CCBP assembled data from over 28 000 children aged 4–17 years of European ancestry across 22 studies. We conducted the largest GWAS meta-analyses (meta-GWAS) to date of four BP traits—SBP, diastolic BP (DBP), pulse pressure (PP), and mean arterial pressure (MAP)—and resting HR in childhood, and replicated significant single-nucleotide polymorphisms (SNPs) in children of European (*n* = 1669), African-American (*n* = 835), and East-Asian ancestries (*n* = 1114). Then we performed comprehensive follow-up analyses, including comparisons with adult GWAS findings, polygenic risk score (PRS) analyses in independent cohorts of diverse ancestries, and a phenome-wide association study (PheWAS) in the UK Biobank. We aimed to (i) identify genetic loci associated with these traits in children, (ii) quantify the shared and distinct genetic architecture between childhood and adulthood, and (iii) investigate associations between PRSs derived from childhood BP and HR GWAS and adult disease outcomes using PheWAS. Our study provides novel insights into the genetic regulation of cardiovascular traits in early life and offers new evidence linking childhood genetic risk to adult health outcomes.

## Methods

### Study design and population

**Figure 1:**
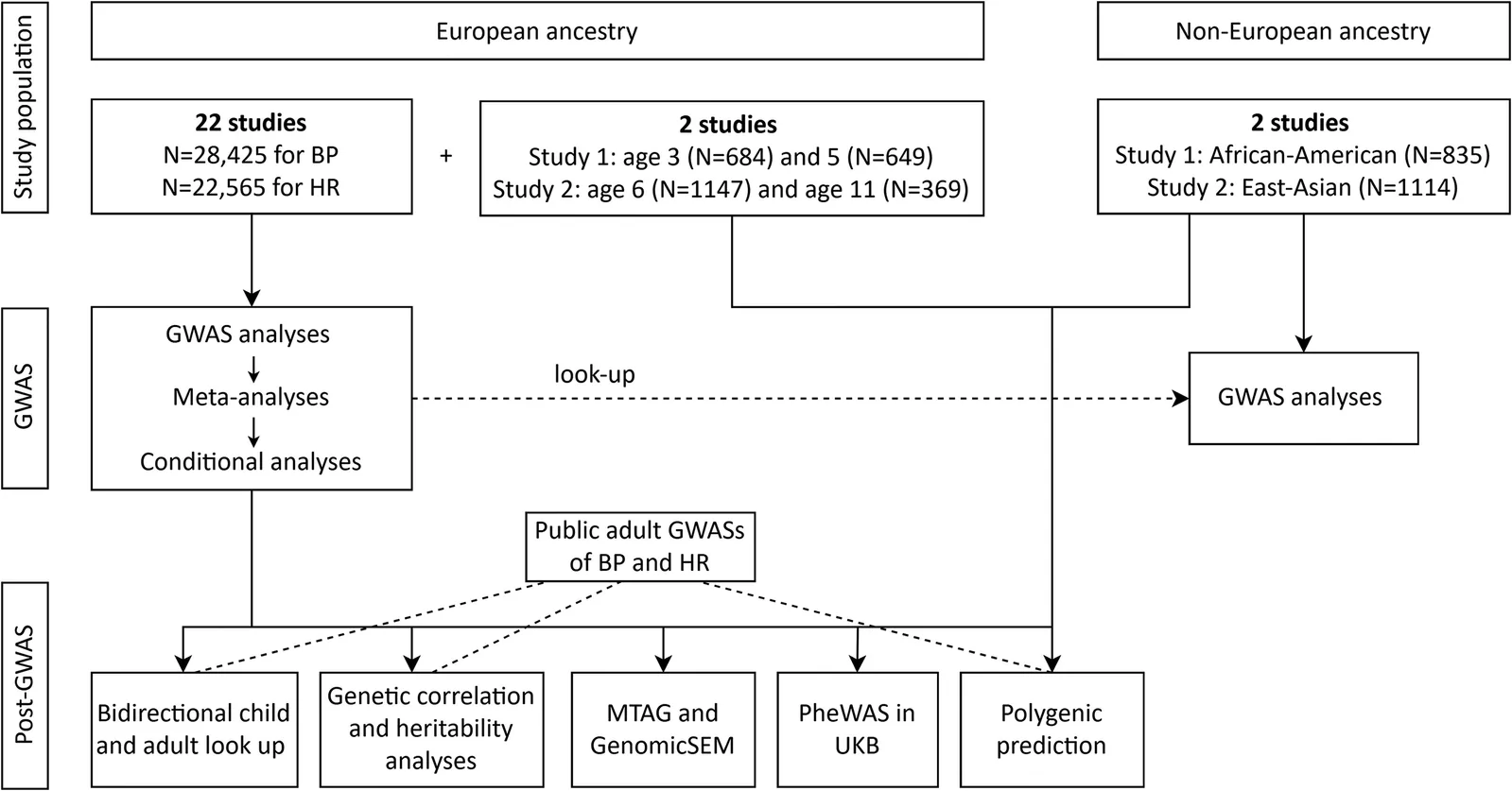
Overview of the study design. BP, blood pressure; HR, heart rate; PheWAS, phenome-wide association study; GWAS, genome-wide association study; UKB, UK Biobank

An overview of the study design is presented in *Figure 1*. We conducted meta-analyses of GWAS for BP traits in 28 425 children of European ancestry across 22 studies (see Supplementary data online, *Table S1*). Among these, we performed a separate meta-analysis of GWAS for HR in 22 565 children from 16 studies with available HR data. We also conducted GWASs of BP and HR for replication and PRS analyses in two independent European-ancestry cohorts (EDEN^24^ and the Amsterdam Born Children and their Development cohort [ABCD],^25^) and two non-European cohorts: African-American children from the Georgia Prevention Institute (GPI)^26^ and East-Asian children from the Tohoku Medical Megabank Project Birth and Three-Generation Cohort Study (TMM BirThree).^27^ In this study, we define children as individuals younger than 18 years, acknowledging that those aged 12–17 years may also be referred to as adolescents.

Detailed information on these 26 studies is presented in Supplementary data online, *Table S1*. Most studies measured SBP, DBP, and HR using a digital automatic BP monitor with an appropriate cuff size in sitting or supine position and used the average of two or three repeated measurements. One cohort used a manual sphygmomanometer, and another cohort measured 24-h ambulatory BP. Details of outcome measurements and characteristics for BP traits and HR in each study are presented in Supplementary data online, *Table S2*. All studies were approved by the ethics committees of the respective institutions.

### Meta-analyses of GWASs

#### Study-level analyses

Each study performed GWASs for SBP, DBP, PP, MAP, and HR using a standardized analysis plan. PP was calculated as the difference between SBP and DBP, and MAP was calculated as 1/3rd of SBP plus 2/3rd of DBP. Residuals were generated from linear regression models adjusted for age, sex, height, and genetic principal components (PCs) to remove variation attributable to non-genetic demographic factors, growth and population structure, followed by rank-based inverse normal transformation. Linear regression under an additive genetic model, with genotypes coded as zero, one, or two copies of the effect allele, was conducted to test association between each genetic variant and the adjusted phenotype using GCTA,^28^ MACH2QTL,^29^ PLINK2,^30^ ProbAbel,^31^ SNPTEST,^32^ and SAIGEgds.^33^ Details of genotyping, quality control, imputation, and statistical methods in each study are provided in Supplementary data online, *Table S3* and Supplementary Methods. Summary statistics underwent harmonized quality control using the GWASInspector R package^34^ to generate cleaned GWAS files ready for meta-analysis (for more details see Supplementary data online, *Table S4* and Supplementary Methods).

#### Meta-analysis

Fixed-effects inverse-variance weighted meta-analyses were performed in METAL,^35^ pooling per-allele effect size estimates (beta coefficients) and their corresponding standard errors across studies. Genomic control was applied to studies with lambda larger than 1 (see Supplementary data online, *Table S4*). SNPs were included in the final meta-analysis if present in at least one-third of all studies or total sample size. The final datasets consisted of over 8 million SNPs per trait. Between-study heterogeneity was assessed and reported using standard heterogeneity statistics (I^2^ and *P*-values) as implemented in METAL.

Independent loci were defined using FUMA’s SNP2GENE module^36^ with linkage disequilibrium (LD) clumping^37^ (±250 kb window, LD r^2^ < 0.1, where r^2^ reflects the correlation between pairs of markers), and lead SNPs were defined as the most significant variant in each region. SNPs with *P*-value < 5 × 10^−8^ were considered genome-wide significant. Conditional analysis was performed using GCTA-COJO^38^ with a 5 Mb LD window to identify independent SNPs within loci (i.e. secondary SNPs). Novel SNPs were those >1 Mb from known loci and not in LD (r^2^ < 0.1) with previously reported variants.

#### Annotation of variant functions

SNPs were annotated using SNPAnnotator,^39^ which provided variant classification (e.g. exonic, intronic) and functional prediction using combined annotation-dependent depletion (CADD) scores.^40^ SNPs with a CADD score ≥ 10 or ≥ 20 were considered among the top 10% and top 1% most deleterious. GWAS Catalog^41^ (version: e108 r2023-01-30) was used to identify the reported associated traits or outcomes with lead and secondary SNPs and their linked variants (within 1Mb of those SNPs and r^2^ ≥ 0.8).

### Replication in European and other ancestries

We looked up associations between lead and secondary SNPs and each trait in four independent replication cohorts. For European ancestry, we conducted GWAS in the EDEN cohort (*n* = 522 at age 5) and ABCD cohort (*n* = 1147 at age 6) using linear regression under an additive genetic model. Results from these two cohorts were then combined through fixed-effect meta-analysis to provide replication evidence in European children (total *n* = 1669). Replication analyses were also conducted in 835 African-American children from the GPI cohort and 1114 East-Asian children from the TMM BirThree Cohort Study using the same analytical framework. Replication was defined as a two-sided *P*-value <.05 with concordant direction of association, assessed based on the sign of the effect estimate.

### Comparison with adult GWAS results

We compared lead and secondary SNPs in the childhood meta-analyses with findings from large adult GWASs of BP (*n* = 1 028 980)^7^ and HR (*n* = 835 464),^15^ and vice versa. GWAS results on MAP available in the UK Biobank European individuals (*n* = 417 001) were used.^42^ Significance was defined as a two-sided *P* value < .05 with a consistent direction of association based on the sign of the effect estimate. To examine whether the adult BP and HR GWAS loci that were also significant in childhood (i.e. shared SNPs) had distinct effect sizes or biological features, we compared effect sizes between shared and non-shared SNPs using Wilcoxon rank-sum tests and conducted gene-set enrichment analysis using FUMA^36^ for each subset of loci.

### Genetic correlation and heritability analyses

LD score regression (LDSC)^43^ was used to calculate genetic correlations (r_g_) between childhood and adulthood BP traits^7^ and HR^15^ and estimate SNP-based heritability. Cross-trait LDSC models were fitted to quantify r_g_ (i) among childhood BP and HR traits, (ii) between childhood and adulthood BP/HR traits, and (iii) among adulthood BP and HR traits. LD scores were computed from the 1000 Genomes^37^ European data, and only HapMap3 SNPs were included to ensure high imputation quality. An r_g_ = 0 means that two traits being analysed are influenced by independent genetic factors, and an |r_g_| = 1 means that the genetic factors of two traits completely overlap.

### Polygenic prediction

We constructed genetic risk scores (GRSs, using genome-wide significant SNPs) and polygenic risk scores (PRSs, using full summary statistics via PRS-PCA^44^) based on both childhood and adult GWASs of BP^7^ and HR^15^:

- childhood-based GRSs of genome-wide significant SNPs identified from our current meta-GWAS of childhood BP traits and HR;
- childhood-based PRSs using our current summary statistics of childhood BP traits and HR meta-GWAS;
- adult-based GRSs of genome-wide significant SNPs identified from previously published meta-GWAS of adulthood BP traits^7^ and HR^15^;
- adult-based PRSs using summary statistics of adulthood BP traits^7^ and HR^15^ meta-GWAS.

These scores were evaluated in the EDEN cohort (*n* = 574 at age 3 and *n* = 522 at age 5), the ABCD cohort (*n* = 1147 at age 6 and *n* = 369 at age 11), TMM BirThree Cohort Study (divided into two sub cohorts: *n* = 572 with genotyping chip JPAv2 and *n* = 542 with chip NEO, mean age 10.2 years with age range from 7 to 17 years), and GPI cohort (*n* = 835, mean age 14.7 years with age range from 4 to 17 years). The proportion of phenotypic variance explained, and quintile-based risk stratification were assessed. SNPs included in the childhood-based GRSs are displayed in Supplementary data online, *Table S5*. More details are provided in the Supplementary Methods.

### Phenome-wide association study in the UK Biobank

PheWAS analyses were performed to investigate the associations between childhood-based PRSs of BP and HR with 178 health-related phenotypes from the UK Biobank,^45^ including 89 binary and 89 continuous phenotypes. Binary disease phenotypes for the PheWAS were generated using the R package ukbpheno^46^ which integrates clinical codes and self-reported data fields to provide harmonized, well-defined outcomes. To ensure statistical power and clinical relevance, we restricted analyses to traits with prevalence >1% and to major, well-recognized diseases, resulting in 89 binary outcomes spanning multiple health domains, with emphasis on cardiometabolic diseases (see Supplementary data online, *Table S6*). Binary outcomes were analysed using logistic regression to estimate odds ratios (OR), and continuous traits were modelled using linear regression, consistent with standard practice in large-scale PheWAS. To enable direct comparison of effect sizes across traits, all continuous phenotypes were transformed using rank-based inverse normalisation prior to analysis. Age, age^2^, sex, genotyping chip, and the first ten genetic principal components were included as covariates. To examine whether early-life genetic susceptibility was associated with later-life outcomes independently of adult genetic susceptibility, we additionally adjusted for the adult BP PRS in the PheWAS analyses. As the UK Biobank was included in the latest adult BP GWAS, adult BP-PRSs were calculated using ICBP-only BP GWAS summary statistics (*n* = 299 024 from 77 studies)^7^ to avoid sample overlap. The corresponding adult BP-PRS was then included as a covariate in each regression analysis. BP values in the UK Biobank were adjusted for any antihypertensive medication use in summary (+15 mmHg for SBP, +10 mmHg for DBP).^47^ To control false positives and prioritize robust associations, Bonferroni-adjusted *P* values were computed as raw *P* values × 178, and associations were considered significant at adjusted *P*-value < .05. Analyses were performed using STATA (version 16). This study was conducted under application number 74 395 from the UK Biobank Resource.

### Multi-trait GWAS analysis

We applied GenomicSEM^48^ and MTAG^49^ jointly analysing BP traits and HR for additional locus discovery. Details are provided in the Supplementary Methods.

## Results

### Meta-analysis results

**Figure 2:**
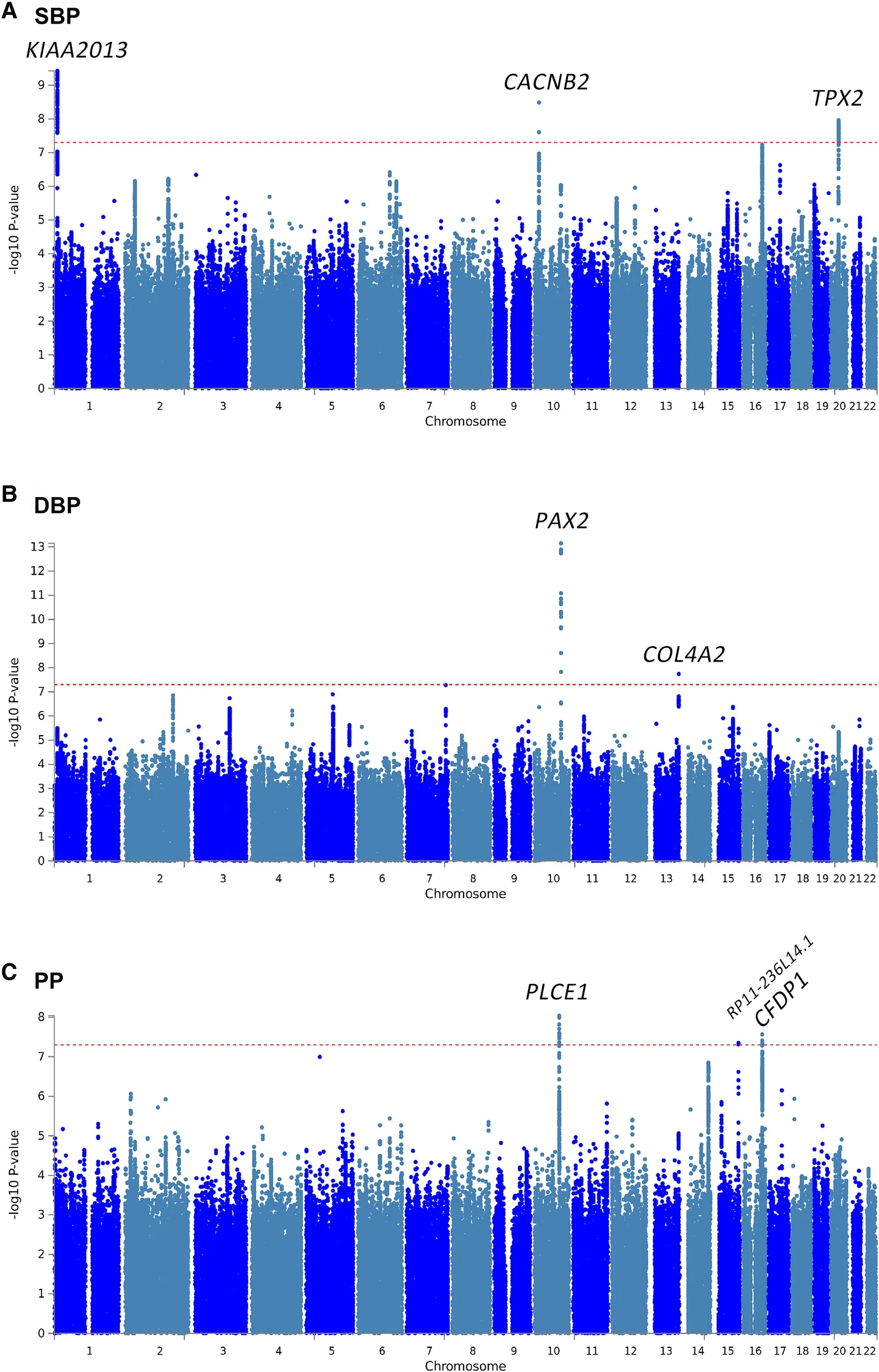
Manhattan plot of genome-wide associations for systolic, diastolic and pulse pressure. (A) Systolic blood pressure (SBP), (B) diastolic blood pressure (DBP), and (C) pulse pressure (PP). The x-axis represents the genome in physical order; the y-axis shows −log_10_  *P*-values for individual single-nucleotide polymorphism (SNP) associations with blood pressure traits from the meta-analyses in European children (*n* = 28 425). Genome-wide significance at *P*-value < 5 × 10^−8^ is indicated by the broken line. Lead SNPs are annotated with the nearest genes. DBP, diastolic blood pressure; PP, pulse pressure; SBP, systolic blood pressure

**Figure 3:**
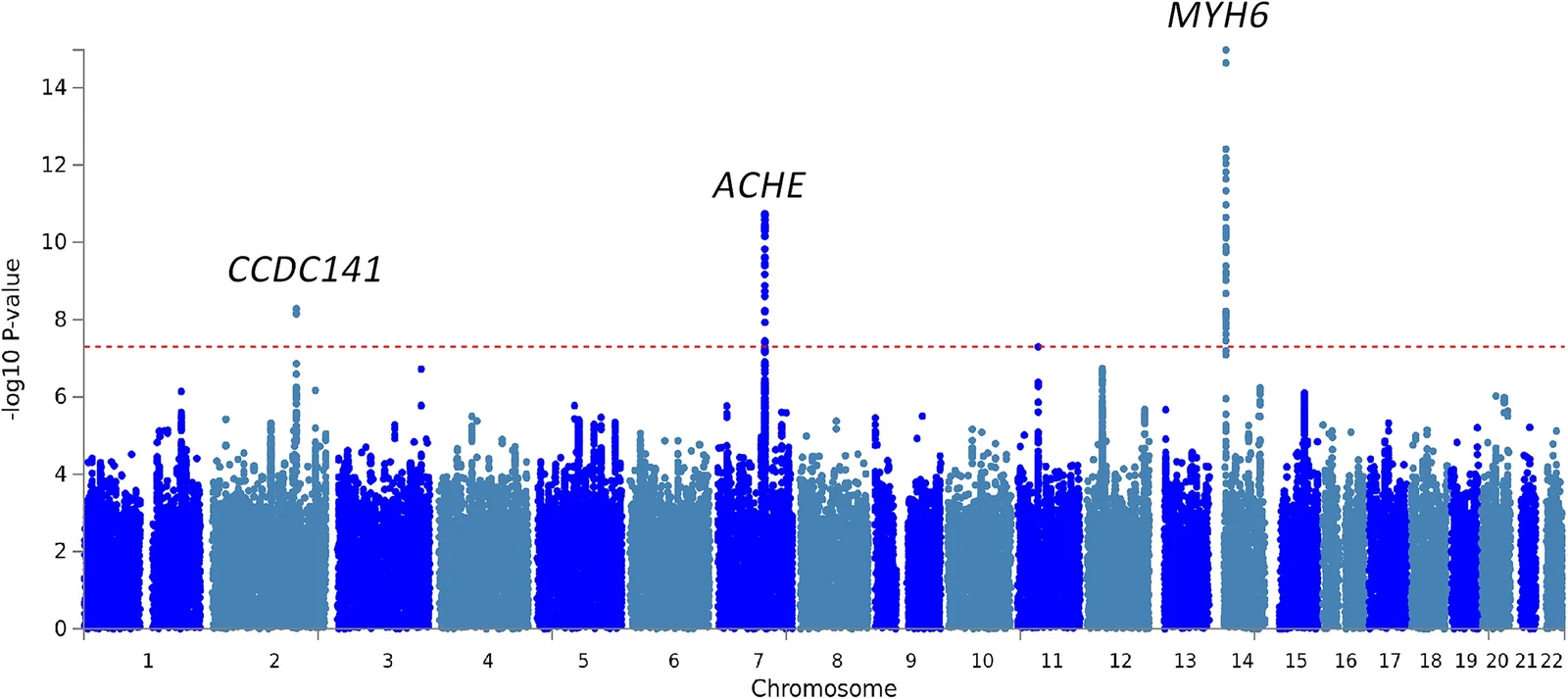
Manhattan plot of genome-wide associations for HR. The x-axis represents the genome in physical order; the y-axis shows −log_10_  *P*-values for individual single-nucleotide polymorphism (SNP) associations with heart rate (HR) from the meta-analyses in European children (*n* = 22 565). Genome-wide significance at *P*-value < 5 × 10^−8^ is indicated by the broken line. Lead SNPs are annotated with the nearest genes

We performed meta-analyses in 28 425 children of European ancestry from 22 studies for BP traits, and in 22 565 children from 16 studies for HR. We identified eight independent loci genome-wide significantly associated with childhood BP traits (*Table 1*): three loci for SBP, two for DBP, and three for PP, with no overlap across these BP traits. Four MAP loci overlapped with SBP or DBP (see Supplementary data online, *Table S7*). Three independent loci reach genome-wide significance for childhood HR (*Table 1*). Manhattan plots are shown in *Figure 2* for SBP, DBP, and PP, *Figure 3* for HR, and Supplementary data online, *Figure S1* for MAP. No evidence of inflation was observed (LDSC intercepts: 0.996–1.007, QQ plots in Supplementary data online, *Figure S2*). Regional plots for lead SNPs are shown in Supplementary data online, *Figure S3*. Conditional analysis identified one additional independent SNP for PP (rs12773803, *NOC3L*) (see Supplementary data online, *Table S8*).

**Table 1:** Variants associated with SBP, DBP and PP (22 studies) and HR (16 studies) at genome-wide significance in meta-analyses of children of European ancestry

Locus	RSID	Chr	Position (hg19)	Nearest gene	Function	Trait	EA	OA	EAF	Beta	SE	*P* value	I^2^_HET_	P_HET_	N_TOTAL
1) Blood pressure traits															
1	rs113573379^a^	1	11 977 327	*KIAA2013*	intergenic	SBP	A	C	0.047	−0.1291	0.0206	3.77 × 10^−10^	0	0.512	27 381
2	rs12258967	10	18 727 959	*CACNB2*	intronic	SBP	G	C	0.281	−0.0585	0.0099	3.26 × 10^−9^	8.4	0.350	27 678
3	rs79958663	10	95 910 761	*PLCE1*	intronic	PP	T	C	0.160	−0.0677	0.0118	9.20 × 10^−9^	3.3	0.416	28 423
4	rs11190709	10	102 552 663	*PAX2*	intronic	DBP	A	G	0.889	0.1042	0.0139	7.15 × 10^−14^	22.8	0.173	27 455
5	rs872588	13	111 015 877	*COL4A2*	intronic	DBP	T	C	0.406	−0.0534	0.0095	1.81 × 10^−8^	38.9	0.040	24 031
6	rs4984497	15	96 635 899	*RP11-236L14.1*	intergenic	PP	C	T	0.680	0.0507	0.0093	4.46 × 10^−8^	0	0.778	28 423
7	rs1011121	16	75 325 933	*CFDP1*	intergenic	PP	G	A	0.585	0.0488	0.0088	2.72 × 10^−8^	12.9	0.291	27 576
8	rs6060954	20	30 383 187	*TPX2*	intronic	SBP	T	G	0.301	−0.0545	0.0095	1.09 × 10^−8^	31.6	0.088	27 416
2) Heart rate															
1	rs142556838	2	179 747 068	*CCDC141*	intronic	HR	T	C	0.083	0.103	0.0176	5.23 × 10^−9^	47.6	0.021	21 783
2	rs17884589	7	100 493 711	*ACHE*	UTR5	HR	A	G	0.189	0.083	0.0123	1.80 × 10^−11^	30	0.130	21 964
3	rs445754	14	23 863 802	*MYH6*	intronic	HR	T	G	0.222	0.0939	0.0117	1.05 × 10^−15^	45.8	0.024	22 565

Chr indicates chromosome; DBP, diastolic blood pressure; EA, effect allele; EAF, effect allele frequency; HET, heterogeneity; HR, heart rate; OA, other allele; PP, pulse pressure; SBP, systolic blood pressure; SE, standard error; UTR5, 5′ untranslated region. Italic text indicates gene names. P_HET_ was derived from a Cochran’s Q-test (two-sided) for heterogeneity.

^a^rs113573379 is in LD with rs79493618 (lead SNP for MAP) (r^2^= 0.86).

All lead and secondary SNPs were located in non-coding regions (*Table 1*). Among SNPs in high LD (r^2^ ≥ 0.8) with lead and secondary SNPs, four were non-synonymous: two in genes *PLCE1* and *NOC3L* for PP and two in *CCDC141* and *UFSP1* for HR. Three showed a high CADD score of 10 or greater, indicating a potentially pathogenic effect (CADD = 23.8 for *PLCE1*, 25 for *CCDC141*, and 12.01 for *UFSP1*) (see Supplementary data online, *Table S9*). The GWAS catalogue search showed that 9 out of 10 SNPs (or linked SNPs; r^2^ ≥ 0.8) for BP traits were also associated with other traits (see Supplementary data online, *Table S10*).

### Replication in European and other ancestries

Associations of lead and secondary SNPs were evaluated in European, African-American and East-Asian children (see Supplementary data online, *Table S11*). One DBP/MAP SNP (rs11190709 in *PAX2*), one MAP SNP (rs79493618 in *RP5-934G17.6*), one PP SNP (rs4984497 in *RP11-236L14.1*), and two HR loci (rs142556838 in CCDC141, rs445754 in *MYH6*) were replicated in European children. Two PP lead SNPs (rs79958663 in *PLCE1* and rs1011121 in *CFDP1*) were replicated in African-American children, and one HR lead SNP (rs445754, *MYH6*) was replicated in East-Asian children.

### Comparison with adult GWAS results

Nine out of 10 SNPs for BP traits (but not rs872588 in *COL4A2*) and all three SNPs for HR were concordant with adult GWAS results (*P* < .05 and same direction of effect size, Supplementary data online, *Table S12*). Rs872588 (*COL4A2)* was not significant in adults (*P* = .58) and may be age-specific, although it lies near *COL4A2*, a previously reported adult BP locus.^6^ Furthermore, eight SNPs for BP traits (except rs12773803 in *NOC3L*) and all three SNPs for HR also achieved genome-wide significance in the adult GWAS. Notably, rs12773803 (secondary SNP for PP, *P* = 1.22 × 10^−6^ in adult PP GWAS) is in high LD with the adult BP locus (lead SNP rs12781628, r^2^ = 0.98) reported by Surendran *et al*.^50^ both mapping to the same nearest gene, *NOC3L*.

Reverse lookups identified 152 (15.3%) SNPs for SBP, 132 (14.2%) for DBP, 188 (15.2%) for MAP, 78 (9.7%) for PP, and 90 (21.3%) for HR that were also significant in childhood meta-GWAS results (*P* value < .05 and same direction of effect size, Supplementary data online, *Table S13A*–13*E*). Effect sizes between adults and children were moderately correlated (r = 0.53 for SBP, r = 0.58 for DBP, r = 0.46 for MAP, r = 0.39 for PP, r = 0.72 for HR; Supplementary data online, *Figure S4*). Shared adult BP and HR loci showed significantly larger effect sizes than non-shared loci (Wilcoxon *P* < .05 for all traits; Supplementary data online, *Figure S5*). FUMA analyses revealed that shared BP loci were enriched in G protein-coupled receptor (GPCR) signalling and sensory signalling pathways, whereas adult-specific loci were enriched in developmental, vascular, and chromatin-regulatory processes (see Supplementary data online, *Table S13F* and *13G*). Both shared and adult-specific HR loci converged on cardiac conduction and contraction pathways (see Supplementary data online, *Table S13H* and *13I*), underscoring the physiological continuity of HR regulation across the life course.

### Genetic correlations between childhood and adulthood

**Figure 4:**
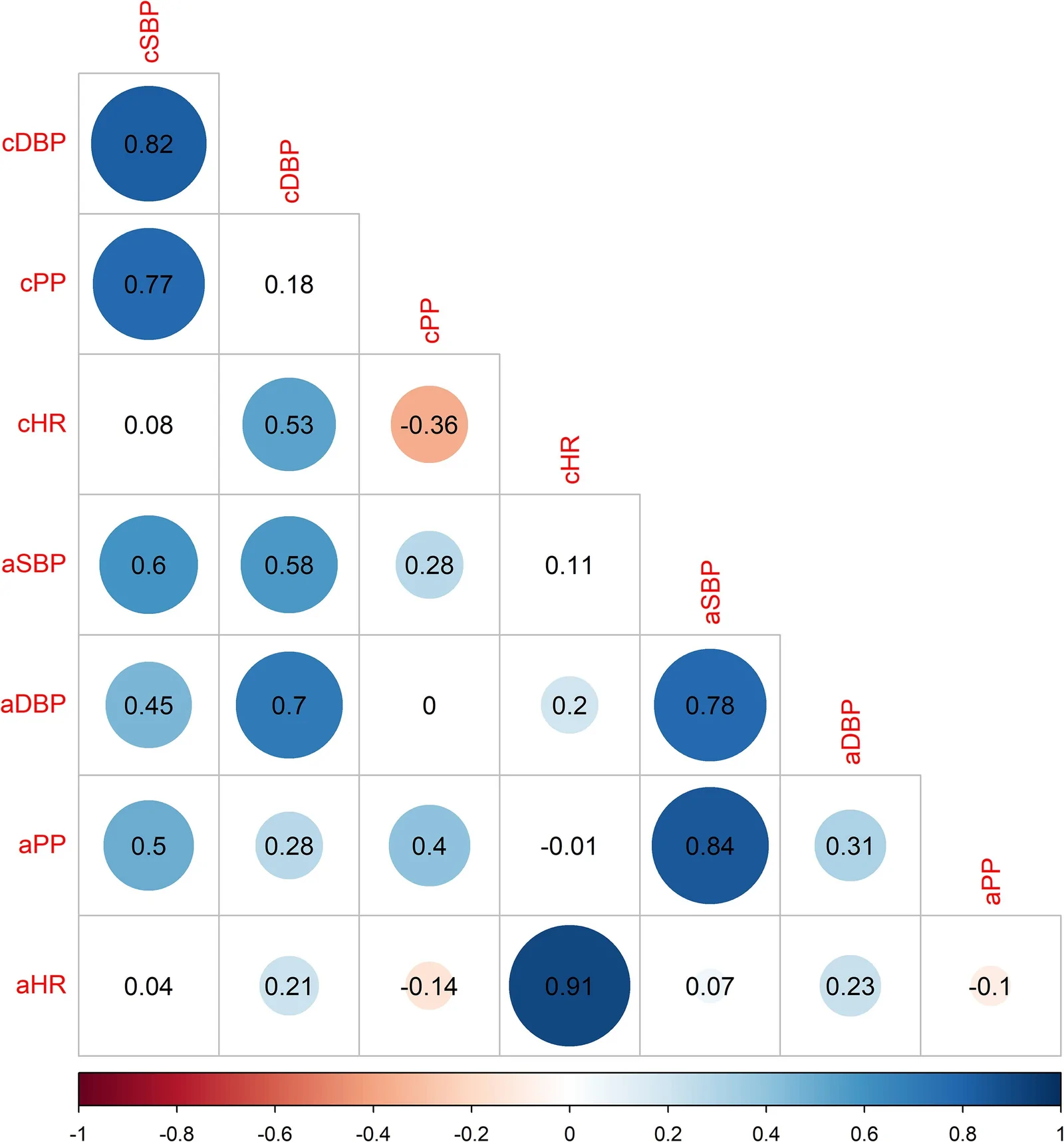
Genome-wide genetic correlations within and between childhood and adulthood blood pressure traits and heart rate. Numbers represent correlation coefficients, and numbers without circles are non-significant coefficients. The 95% confidence intervals and exact *P*-values are provided in Supplementary data online, *Table S14*. aDBP, adulthood diastolic blood pressure; aHR, adulthood heart rate; aPP, adulthood pulse pressure; aSBP, adulthood systolic blood pressure; cDBP, childhood diastolic blood pressure; cHR, childhood heart rate; cPP, childhood pulse pressure; cSBP, childhood systolic blood pressure

Genome-wide genetic correlation analyses (*Figure 4*, Supplementary data online, *Table S14*) showed moderate correlations (r_g_ [95% confidence interval]) between childhood and adulthood BP traits: r_g_ = 0.60 [0.49–0.70] for SBP, 0.70 [0.57–0.83] for DBP and 0.40 [0.29–0.51] for PP). For HR, the genetic correlation with adulthood was near unity (r_g_ = 0.91 [0.66–1.16]).

### SNP-based heritability and polygenic prediction results

SNP-based heritability estimates in childhood were 12.7% (*P*-value = 1.28 × 10^−11^) for SBP, 10.5% (*P*-value = 2.80 × 10^−9^) for DBP, 12.0% (*P*-value = 4.14 × 10^−11^) for MAP, 9.7% (*P*-value = 1.12 × 10^−6^) for PP and 7.8% (*P*-value = 9.07 × 10^−4^) for HR. For comparison, corresponding SNP-based heritability estimates in adults were 12.3% for SBP, 11.3% for DBP, 11.0% for PP, and 10.8% for HR, suggesting broadly similar magnitudes of common genetic contribution between childhood and adulthood.

**Figure 5:**
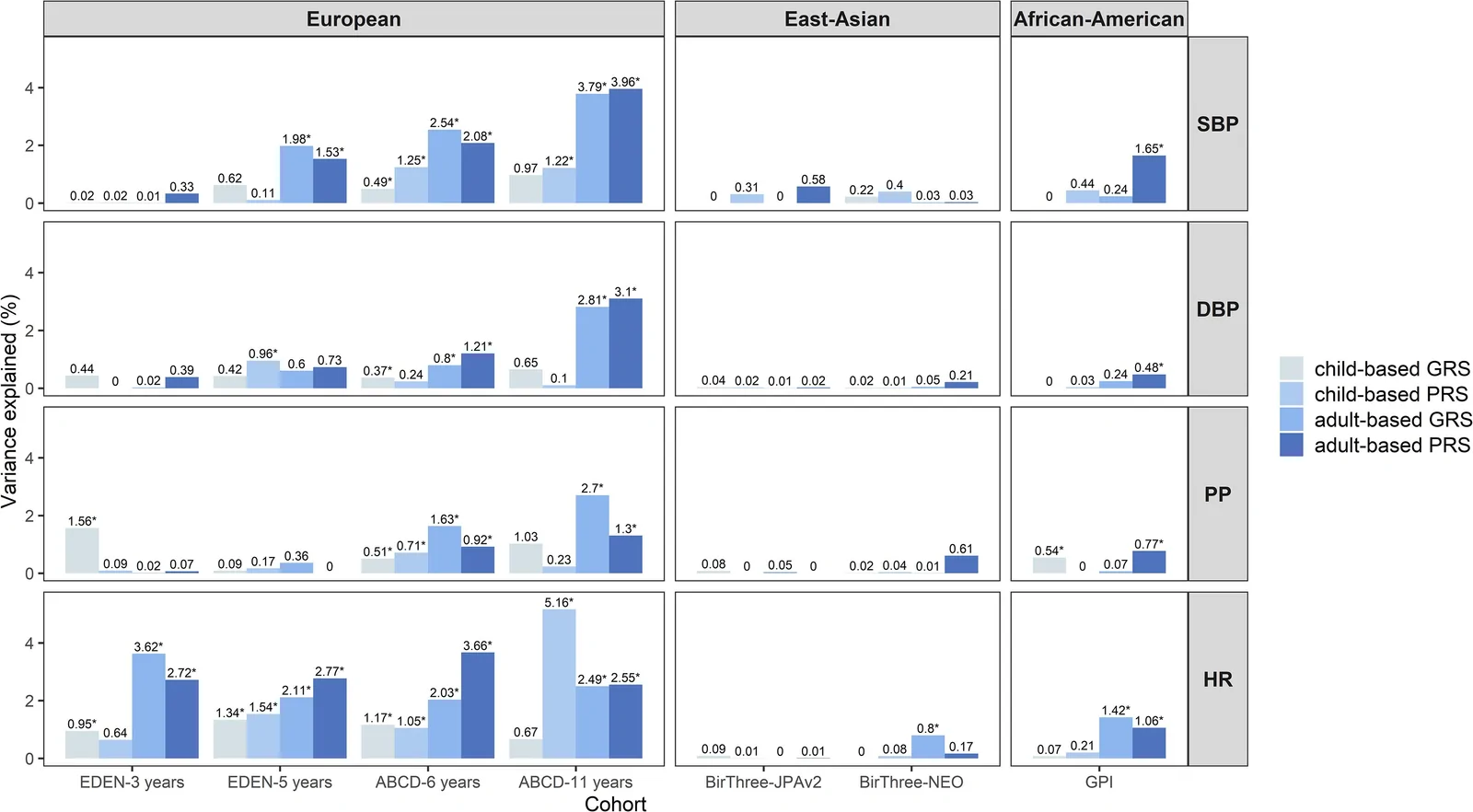
Variance explained by childhood-based and adult-based genetic and polygenic risk scores in cohorts of three ancestries. Including two cohorts of European children (EDEN cohort: *n* = 574 at age 3 and *n* = 522 at age 5 and Amsterdam Born Children and their Development (ABCD) cohort: *n* = 1147 at age 6 and *n* = 369 at age 11), a cohort of East-Asian children (TMM BirThree Cohort Study, divided into two sub cohorts: *n* = 572 with genotyping chip JPAv2 and *n* = 542 with chip NEO, mean age 10.2 years with age range from 7 to 17 years) and a cohort of African-American children (GPI, *n* = 835, mean age 14.7 years with age range from 4 to 17 years). DBP, diastolic blood pressure; HR, heart rate; PP, pulse pressure; SBP, systolic blood pressure. *P < .05. The exact *P* values are provided in Supplementary data online, *Table S15A*

Variances explained by GRSs and PRSs are shown in *Figure 5* and Supplementary data online, *Table S15A*. These scores performed best in European children, where adult-based scores generally explained more variance than childhood-based scores. In addition, variance explained by adult-based scores showed an increasing trend with age for BP traits but remained relatively stable for HR. Childhood-based GRSs, although based on a limited number of SNPs, explained a small but significant proportion of variance at some ages (e.g. 0.49% for SBP, 0.37% for DBP, 0.51% for PP, 1.17% for HR at 6 years). Childhood-based PRSs based on full summary statistics explained up to 1.6% of BP variance and 5.2% for HR. Quintile analyses showed significant differences in BP and HR across PRS quintiles, but only in European children (see Supplementary data online, *Figure S6* and Supplementary data online, *Table S15B*).

### PheWAS results

**Figure 6:**
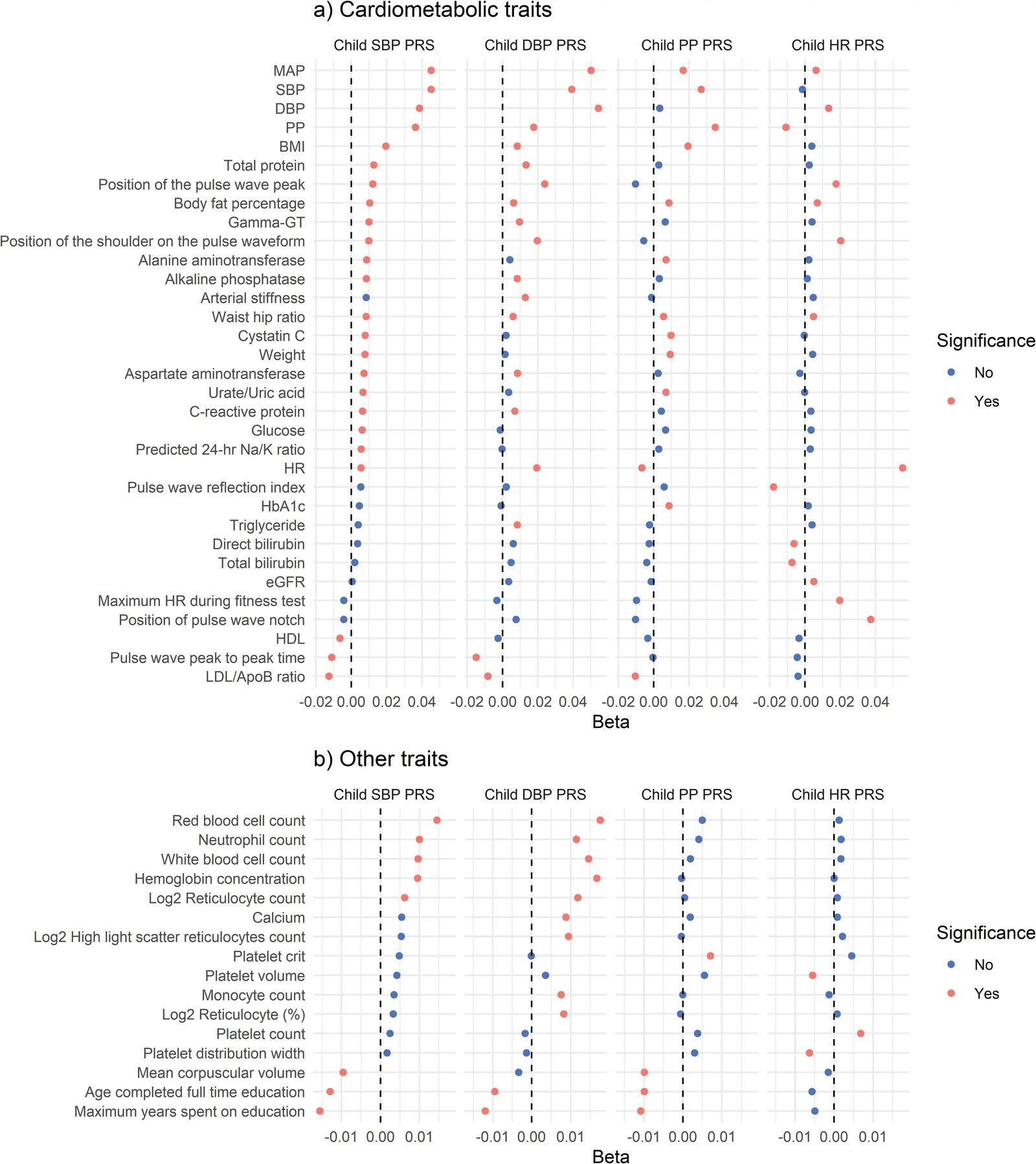
Significant associations between child blood pressure and heart failure polygenic risk scores with continuous traits in UK Biobank adults. (a) Cardiometabolic traits, (b) Other traits. All continuous traits were inverse rank normalized. Associations were considered significant at a Bonferroni-adjusted *P*-value < .05. Point estimates (Betas) are shown without confidence intervals, as statistical inference is based on Bonferroni-adjusted *P*-values rather than confidence intervals including zero. Detailed PheWAS results are provided in Supplementary data online, *Table S16A*. DBP, diastolic blood pressure; HDL, high-density lipoprotein; HR, heart rate; LDL, low-density lipoprotein; MAP, mean arterial pressure; PP, pulse pressure; Predicted 24-hr Na/K ratio, predicted 24-h urinary sodium to potassium ratio; PRS, polygenic risk score; SBP, systolic blood pressure

**Figure 7:**
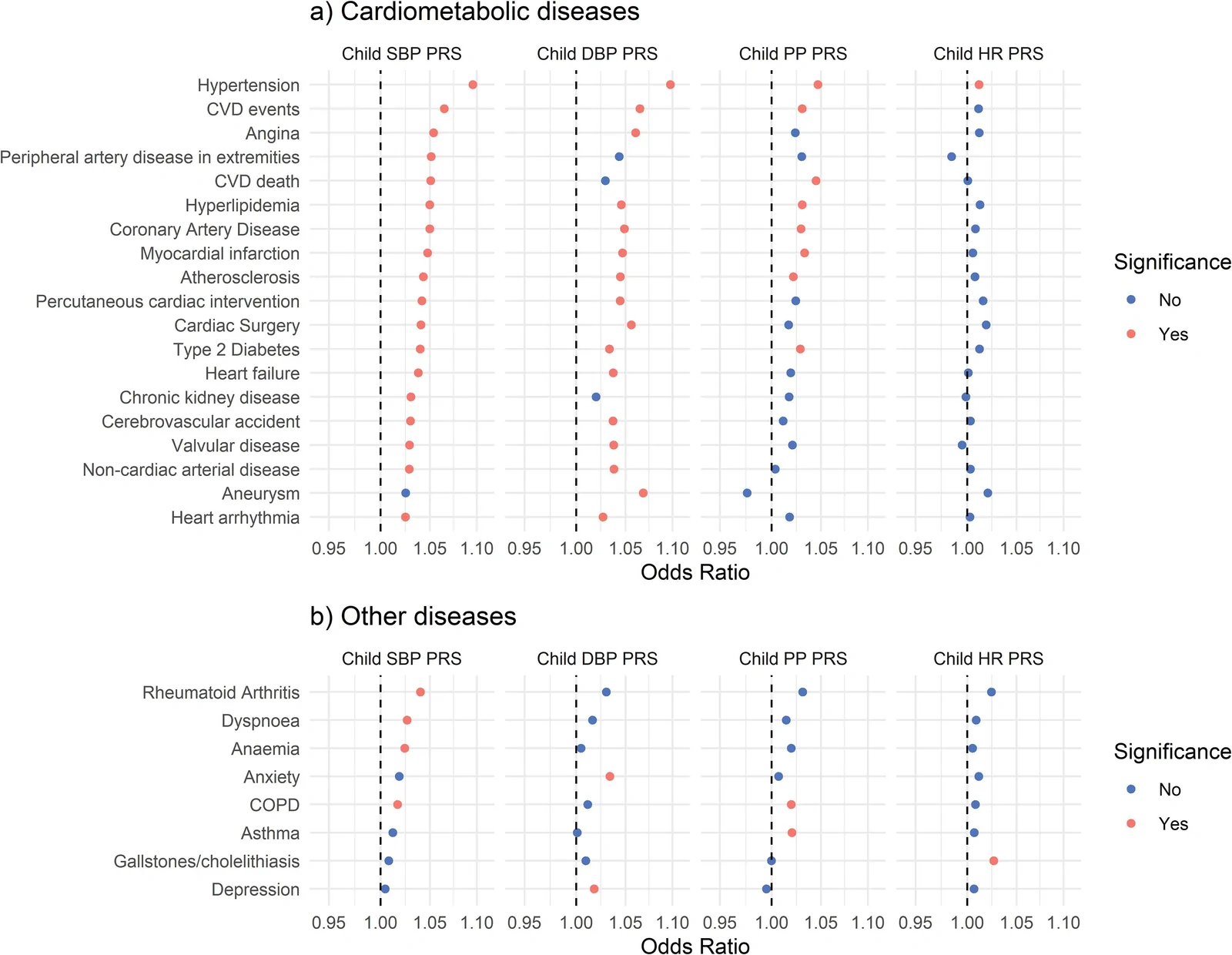
Significant associations between child blood pressure and heart failure polygenic risk scores with diseases in UK Biobank adults. (a) Cardiometabolic diseases, (b) Other diseases. Associations were considered significant at a Bonferroni-adjusted *P*-value < .05. Point estimates (odds ratios) are shown without confidence intervals, as statistical inference is based on Bonferroni-adjusted *P*-values rather than confidence intervals including one. Detailed PheWAS results are provided in Supplementary data online, *Table S16B*. DBP, diastolic blood pressure; COPD, chronic obstructive pulmonary disease; CVD, cardiovascular disease; HR, heart rate; PP, pulse pressure; PRS, polygenic risk score; SBP, systolic blood pressure

A total of 457 418 European UK Biobank adults (mean age: 57.3 ± 8.02 years, 54.3% female) were included in the PheWAS analyses (flowchart in Supplementary data online, *Figure S7*). Supplementary data online, *Table S16A* presents the PheWAS results for all 89 continuous phenotypes, while Supplementary data online, *Table S16B* displays results for all 89 binary phenotypes. Among 178 phenotypes investigated, child BP PRSs were significantly associated with 66 phenotypes, while child HR PRS was associated with 19 phenotypes after Bonferroni correction (adjusted *P*-value <.05) (see Supplementary data online, *Table S16A* and *16B*, *Figures 6* and *7*). Both child BP and HR PRSs showed strong associations with their corresponding adult traits. Notably, child BP PRSs were also linked to various cardiometabolic outcomes (e.g. adiposity markers, lipid profiles, liver function markers). Specifically, higher child SBP PRS values were associated with an increased risk of numerous cardiometabolic diseases (*Figure 7*), such as hypertension (OR 1.10 per SD of child SBP PRS), type 2 diabetes (OR 1.04), hyperlipidaemia (OR 1.05), angina (OR 1.05), myocardial infarction (OR 1.05), heart failure (OR 1.04), heart arrhythmia (OR 1.02), cerebrovascular event (OR 1.03), peripheral artery disease (OR 1.05), chronic kidney disease (OR 1.03), and cardiovascular disease-related mortality (OR 1.05). In contrast, child HR PRS was only significantly associated with hypertension (OR 1.01). Additionally, child BP PRSs were linked to diverse non-cardiovascular phenotypes, including haematological (e.g. neutrophil counts, anaemia), mental (e.g. anxiety), musculoskeletal (e.g. rheumatoid arthritis), respiratory (e.g.chronic obstructive pulmonary disease [COPD]) and socioeconomic traits (e.g. maximum years spent on education). After adjusting for adult BP-PRS effects, the effect sizes modestly decreased, but most phenotypes remained significant (see Supplementary data online, *Table S16A* and *S16B*).

### GenomicSEM and MTAG results

A GenomicSEM common factor model included SBP, DBP, and HR (see Supplementary data online, *Figure S8*). SBP and DBP are more closely related to each other than to HR in terms of shared underlying factors. No novel loci were identified from the summary statistic dataset for this latent factor. MTAG identified an additional genome-wide significant SNP for DBP (rs10067798 with nearest gene *TENM2*, *P*-value = 7.61 × 10^−9^, Supplementary data online, *Table S17*), which has been associated with traits like educational attainment, but not with adult BP. This locus may represent a childhood-specific BP signal, but it still needs further replication.

## Discussion

Our findings provide new insights into the genetic architecture of childhood BP and HR, highlighting their potential significance for lifelong cardiometabolic health. In the largest GWAS of childhood BP and the first GWAS of childhood HR to date, we identified eight loci genome-wide significantly associated with childhood BP traits and three loci associated with childhood HR. Although these loci are newly identified in the paediatric population, all have been previously reported in GWAS of adult BP and HR, suggesting that genetic influences on cardiovascular regulation emerge early in life (*Structured Graphical Abstract*).

Notably, two of the identified PP loci (*PLCE1, NOC3L*) and two HR loci (*CCDC141, UFSP1*) include non-synonymous variants in high LD with the lead SNPs, indicating potential functional effects. In particular, identifying *PLCE1* as a locus for childhood PP—a marker of arterial stiffness and vascular ageing—offers valuable insights into early-life vascular physiology. *PLCE1* encodes phospholipase C epsilon 1, an enzyme critically involved in regulating intracellular calcium signalling within vascular smooth muscle cells, thus influencing vascular constriction and arterial elasticity.^51^ Additionally, given its known role in early-onset nephrotic syndrome,^52^ genetic variation in *PLCE1* may also affect renal sodium and fluid handling, contributing indirectly to vascular remodelling and altered PP from childhood onwards. Collectively, genetic variation in *PLCE1* may initiate subtle but persistent alterations in vascular structure or function early in life, predisposing individuals to higher cardiovascular risk across their lifespan. Future research to clarify and validate these mechanistic pathways is warranted. For HR, our chromosome 2 signal at *CCDC141* aligns with adult HR GWAS and maps within a region syntenic to the rat chromosome 3 quantitative trait locus for HR^10^—underscoring conserved biology of cardiac chronotropy across species and developmental stages.

Our study reveals a complex polygenic architecture of childhood BP and HR. The SNP-based heritability estimates for BP traits and HR in children were comparable to those estimated in adults. These estimates confirm that a meaningful proportion of trait variability in childhood is attributable to common genetic variants detectable through GWAS. However, <2% of the variance in childhood BP could be explained by current childhood-derived GRSs or PRSs. This gap underscores the well-known issue of ‘missing heritability’^53^ and suggests that increasing sample sizes will continue to discover more loci primarily with smaller effect sizes, as shown by recent GWASs of height.^54^

Our study provides a systematic comparison between childhood and adult GWAS results. We observed that 10%–20% of SNPs previously associated with adult BP and HR were nominally significant in our childhood GWAS, and adult-based GRSs and PRSs significantly predict childhood BP and HR. These findings are consistent with prior work, showing the potential of applying adult-based genetic predictors at an early age for early prevention^55–58^. In line with this, a recent clinical consensus statement has highlighted the potential value of PRSs for life-course cardiovascular risk stratification when integrated with established clinical risk assessment tools.^59^

For HR, we observed a high genetic correlation of 0.91 between childhood and adulthood, together with relatively stable variance explained and PRS-phenotype associations across ages, suggesting that HR is genetically more stable throughout the course of life. This stability may reflect its underlying physiology: HR is primarily regulated by the cardiac pacemaker system and autonomic pathways, which are largely established early in life and remain relatively stable across developmental stages.^60^

In contrast, BP showed shared yet age-specific genetic influences across the life course. We observed moderate genetic correlations between childhood and adult BP traits (rg = 0.4–0.7), indicating partial but not complete overlap in genetic determinants. This is in line with a longitudinal twin study showing that only ∼60% of genetic influence on BP was shared between childhood and early adulthood, with novel genetic effects emerging during development into adulthood.^18^ One plausible explanation is that hormonal changes after puberty may affect the expression of BP-associated genes. Our pathway analyses further suggest that childhood BP shares its strongest genetic determinants with adulthood BP through neurohumoral signalling pathways, whereas additional loci emerge later in life involving vascular remodelling, cellular growth, and epigenetic regulation, reflecting age-specific adaptation in BP control mechanisms.

Consistent with a comprehensive review by Wang and Snieder,^2^ which concluded that partly different genes affect BP in different periods of life, our study provides, to our knowledge, the first GWAS-based estimation of genetic correlations between childhood and adulthood BP traits. Furthermore, our PRS analyses revealed that adult-based PRSs explained increasing variance with age and showed steeper dose-response gradients across PRS quintiles, suggesting an increasing genetic overlap at older ages.^55^ The slightly divergent trends observed among SBP, DBP, and PP further indicate trait-specific maturational trajectories in their genetic regulation. Moreover, our MTAG results identify a potential childhood-specific BP locus (*TENM2*) not previously implicated in adult studies. As CCBP continues to expand, it holds the promise of discovering additional loci with age-specific effects, potentially offering new insights into early BP regulation. Future work should explore age-varying genetic effects in more detail employing new emerging methods^61,62^ such as meta-regression, and integrating exome and whole-genome sequencing data to characterize rare variants, which may exert larger and potentially developmentally specific effects during early growth.^50^

The observed associations between childhood BP polygenic risk and a broad spectrum of adult cardiometabolic outcomes provide compelling evidence for a potential causal link between elevated BP in early life and increased disease risk later in adulthood. In our PheWAS of over 450 000 adults, higher childhood BP PRSs were associated with increased risks of cardiometabolic diseases, including hypertension, type 2 diabetes, coronary artery disease, heart failure, chronic kidney disease, peripheral artery disease, and cardiovascular mortality. The majority of associations between childhood BP PRSs and adult phenotypes remained significant after adjustment for adult BP-PRS effects, indicating that early-life genetic susceptibility exerts effects on later-life outcomes that are partly independent of adult BP genetic influences. These findings align with Mendelian randomisation studies showing that elevated SBP is a causal risk factor for multiple cardiovascular diseases^63,64^ and with epidemiological studies demonstrating that elevated BP in childhood tracks into adulthood and predicts future cardiovascular events^65–67^.

Importantly, the childhood BP PRS serves as a genetic instrument for a test of the potential effects of childhood BP, unconfounded by postnatal socioeconomic position, lifestyle and health factors, on the risk and pathogenesis of adult cardiometabolic diseases. Moreover, associations observed between childhood BP PRS and non-cardiometabolic outcomes—including chronic respiratory diseases such as COPD, asthma, and dyspnoea, underscore the potential broader systemic implications of early-life BP and warrant further investigation. Specifically, we acknowledge that such PheWAS analyses require future follow-up analyses exploring the potential relevance of weak genetic instrument strength and horizontal pleiotropy. It is also important to formally test the extent to which any effect is mediated via adult BP, as has been done for childhood and adult body mass index (BMI),^68^ to better understand the potential added value of interventions to reduce adverse effects of high BP on future health in childhood vs adulthood. We also note substantial heterogeneity in statistical power across the 178 PheWAS outcomes, reflecting differences in outcome type and case-control distributions. Complete results are therefore provided in Supplementary data online, *Table S16A* and *S16B*. Although conservative significance thresholds were applied to prioritize high-confidence findings, clinical interpretation is guided by the effect size magnitude and direction, rather than statistical significance alone. Finally, to facilitate further research across the full spectrum of phenotypes available in the UK Biobank, we have made the childhood BP and HR summary statistics publicly available.

The major strengths of this study include the largest GWAS of childhood BP traits and the first GWAS of childhood HR to date, substantially increasing the statistical power for genetic discovery. As children rarely take antihypertensive medication, the childhood BP GWAS provides a cleaner picture of the genetic association with ‘true’ BP levels. The full GWAS summary statistics are publicly available, providing a valuable resource for future follow-up and translational studies. The planned expansion of the CCBP Consortium, particularly with non-European cohorts, will further enable larger-scale meta-GWAS to identify additional loci. Moreover, the systematic comparison of childhood and adult GWAS results provided a quantitative assessment of both shared and distinct parts of the genetic architectures across the life course for BP and HR. Furthermore, leveraging childhood BP PRSs as genetic proxies for early-life BP enabled the assessment of their long-term associations with a wide range of adult health outcomes within a single analytic framework.

Several limitations should be acknowledged. First, our study mainly focused on children of European ancestry. Although cohorts of African-American and East-Asian ancestry were incorporated, and both shared and ancestry-specific signals were observed for lead SNPs and PRSs, larger GWAS in non-European paediatric populations are needed to comprehensively characterize the genetic architecture of childhood BP and HR across ancestries. Second, given that girls have substantially lower BP than boys during childhood,^69^ sex-stratified BP GWAS may help determine whether these differences extend to sex-specific genetic effects. Third, GWAS analyses were not adjusted for BMI to avoid potential collider bias,^70^ which limits the ability to determine whether genetic effects on HR and BP were independent of adiposity. Nonetheless, only one BP locus (*PAX2*) showed association with BMI (see Supplementary data online, *Table S10*), and this locus was also reported by adult BP GWAS.

## Conclusion

Our findings advance the understanding of the genetic architecture of childhood BP and HR and provide compelling genetic evidence linking childhood BP to a broad spectrum of adult health outcomes—particularly cardiometabolic conditions—thereby supporting the potential for early-life risk stratification and informing targeted prevention strategies from a young age.

## Supplementary Material

ehag313_Supplementary_Data

## Declarations

### Data Availability

Full GWAS summary statistics of our meta-analyses are publicly available on the GWAS Catalogue website data repository (https://www.ebi.ac.uk/gwas) with data accession codes GCST90797639, GCST90797640, GCST90797641, GCST90797642, GCST90797643 for childhood SBP, DBP, PP, MAP, and HR, respectively.
