# Supplementary material for: Genetic determinants of childhood blood pressure and heart rate in relation to adult health outcomes: the consortium of childhood blood pressure
Source: Eur Heart J. 2026 Apr 27;47(33):4621–37. doi: 10.1093/eurheartj/ehag313 (PMC13533560; doi:10.1093/eurheartj/ehag313)
Supplement: Supplementary file 1 [file ehag313_supplementary_data.zip › SupplementaryMaterials_forEHJ_2nd revised_submitted_clean.docx]

**Supplementary Note**

**Supplementary Methods**

**Meta-analyses of GWASs**

**Sample size.** We conducted meta-analyses of GWAS for BP traits in 28,425 children of European ancestry across 22 studies. Among these, we performed a separate meta-analysis of GWAS for HR in 22,565 children from 16 studies with available HR data. As this study represents a consortium-based GWAS meta-analysis, sample size was determined by the availability of eligible participants across contributing cohorts rather than by a priori sample size calculations.

**Study-level analyses.** Individuals were genotyped using high-density arrays and study-specific quality control (QC) was applied on samples (e.g. low call rate, heterozygosity outliers, sex mismatches, duplicated samples, non-European, relatedness) and SNPs (call rate, minor allele frequency, Hardy-Weinberg disequilibrium). Hardy-Weinberg equilibrium was assessed by comparing observed genotype counts with those expected under equilibrium based on allele frequencies $\left( p^{2} + 2pq + q^{2} \right)$, using chi-squared goodness-of-fit tests, and variants showing significant deviation were excluded. Imputation was then performed using the 1000 Genome^1^, HRC^2^, TOPMed^3^ or cross-imputed reference panel data (Table S3). Residuals were generated from linear regression models adjusted for age, sex, height, and genetic principal components (PCs) to remove variation attributable to non-genetic demographic factors, growth and population structure, followed by rank-based inverse normal transformation to ensure an approximately normal phenotype distribution and reduce the influence of heteroscedasticity and extreme values prior to genetic association testing. Covariates were selected a priori to control for differences in demographic variables (age, sex), growth (height) and population stratification (PCs) on blood pressure in children. Adjustment for population structure was performed using genetic PCs, with the number of PCs determined at the cohort level based on ancestry composition and prior validation within each study (Table S3). Genetic principal components were derived using standard eigen-decomposition without rotation. In cohorts containing siblings, handling of related individuals was determined at the cohort level: studies either retained a single individual per family to ensure independence, or explicitly adjusted genetic relationships using mixed-effect models that incorporate a genetic relationship matrix (GRM), depending on cohort structure and available analytical frameworks (Table S3).

**Study-level QC of GWAS results.** We used the GWASInspector R package^4^ to perform a harmonized QC procedure for every GWAS result and generate cleaned GWAS files ready for meta-analysis. The consistency and integrity of the result files were checked and unusable data, including duplicated variants or variants that missed crucial information, were removed. Variant IDs and alleles were then harmonized across studies and allele frequency concordances were checked against the 1000 Genomes reference data. The correlation was calculated between the reported P-value and expected P-value derived from effect size and standard error. Manhattan plots were inspected to identify spurious associations. Quantile-quantile (QQ) plots were examined and the genomic inflation factor (λ_GC_) was calculated to check for study-level genomic inflation. If some problems were detected during QC, we asked the studies to check and fix the problems until the GWAS results passed the QC procedure. Before meta-analysis, the following criteria were used for filtering the GWAS datasets: 1) minor allele frequency (MAF) >= 1%; and 2) imputation quality >0.3. For some studies, stricter criteria were used to filter variants (e.g. MAF >=3% or imputation quality >0.5) according to QQ plots at different criteria (for more details see Table S4).

**Meta-analysis.** Fixed-effects inverse-variance weighted meta-analyses were performed in European children for BP traits and HR using Metal^5^. For all traits, individual study lambdas before genomic control ranged from 0.982 to 1.087, and genomic control was applied to studies with lambda larger than 1 before the meta-analyses (Table S4). Linkage disequilibrium score regression (LDSC) intercepts were estimated using LDSC^6^ to test if meta-analysis results showed inflation due to cryptic relationships or population structure. The observed LDSC intercepts were 0.9959, 0.9969, 0.9967, 0.9967, and 1.0071 for SBP, DBP, MAP, PP, and HR respectively which indicated no inflation, so no further genomic control correction was applied to the meta-level results.

**Conditional analyses**. Genome-wide joint conditional analysis was performed using GCTA-COJO^7^ with a 5 Mb LD window and a genome-wide significance threshold of 5x10^-8^. A total of 10,210 unrelated European-ancestry samples from Lifelines were used as LD reference^8^. For each of the BP traits and HR, summary statistics were analyzed by chromosome to build a stepwise joint conditional model that selected independently associated secondary SNPs.

**G****enetic correlation and heritability analyses**

LDSC intercepts and slopes were examined to assess the linearity and polygenicity assumptions of LDSC. Intercepts were close to 1 for all traits in both childhood (ranged from 0.996 to 1.007) and adulthood (0.986 to 1.112) analyses, indicating minimal confounding and supporting a linear relationship between association statistics and LD scores. Positive slopes and non-zero SNP-based heritability estimates further supported a polygenic architecture.

**Polygenic prediction**

We generated GRSs and PRSs by multiplying the risk allele dosages for each SNP by its respective effect size as weight, and then summed all SNPs in the score. The GRSs included the same set of SNPs for all three BP traits (SBP, DBP, and PP), i.e. 9 SNPs for childhood-based GRSs and 2103 SNPs for adult-based GRSs, but were weighted by the trait-specific beta coefficients from the GWAS results for childhood and adult SBP, DBP, and PP, respectively. For HR, 3 SNPs were included for childhood-based GRS and 434 SNPS with MAF>=1% were included for adult-based GRS. Summary statistics for all SNPs included in the childhood-based GRSs are displayed in Table S5. The PRSs were generated using the PRS-PCA approach^9^. Multiallelic SNPs, SNPs with ambiguous strands (A/T or C/G) or SNPs with MAF<1% were not included. To obtain an independent set of SNPs, an LD-driven clumping procedure was performed in PLINK (r^2^<0.1, 1000 kb window) using the LD reference panel of 503 European samples from 1000 Genomes phase 3^1^. Scores were constructed at 11 selected P-value thresholds (5×10^-8^, 1×10^-7^, 1×10^-6^, 1×10^-5^, 1×10^-4^, 1×10^-3^, 0.01, 0.05, 0.1, 0.5, 1) and were then standardized using z-score transformations. Finally, principal component analysis (PCA) was performed on these scores without rotation, and the first principal component capturing the maximal shared variance across scores was extracted and subsequently standardized (mean = 0, SD = 1) to generate the final PRS used in association analyses. The PRS-PCA approach avoids the need for choosing the PRS with an optimal P-value threshold and has been shown to be an unbiased and powerful way to index polygenic risk.^1^

To evaluate the percentage of variance explained of BP traits and HR by childhood-based and adult-based GRSs and PRSs, we generated the residuals from a regression of each trait against sex, age, height, and the top ten genetic principal components in each of four cohorts. We then fit a second linear model for the trait residuals with GRS or PRS. The adjusted R^2^ of the second model is the estimation of variance explained by the GRS or PRS.

We also performed quintile analyses to evaluate to what extent childhood-based and adult-based PRS were predictive for BP traits and HR. We divided PRSs into quintiles and ran linear regression with adjustment for the same covariates as variance explained analyses to estimate the mean difference in trait value (mmHg or bpm) relative to the lowest PRS quintile.

## Multi-trait GWAS analysis

**GenomicSEM**^10^**.** Summary statistics for the four BP traits (SBP, DBP, MAP, PP) and HR were munged (i.e., processed and prepared for further analysis) and included using the total sample for the GWAS, a minor allele frequency (MAF) cut off of 1 % and SNPs available in the HapMap3 LD panel. We used the LDSC available within the GenomicSEM package and constructed a one factor model based on the traits. An initial common factor model showed that including MAP and PP did not change the correlations between the latent factor and the single traits, thus we excluded these two and kept SBP, DBP and HR.

**MTAG**^11^. First, we removed the duplicated SNPs and SNPs without rsID in the meta-analyzed results of three correlated traits (i.e., SBP, DBP, and HR). Then, the quality-controlled summary statistics of the three traits were used to perform multi-trait GWAS analyses (MTAG) by applying the default settings. MTAG generated trait-specific effect estimates and p values for each SNP.

**References**

1.	Auton A, Abecasis GR, Altshuler DM, et al. A global reference for human genetic variation. *Nature*. 2015;526(7571):68-74. doi:10.1038/nature15393

2.	McCarthy S, Das S, Kretzschmar W, et al. A reference panel of 64,976 haplotypes for genotype imputation. *Nat Genet*. 2016;48(10):1279-1283. doi:10.1038/ng.3643

3.	Taliun D, Harris DN, Kessler MD, et al. Sequencing of 53,831 diverse genomes from the NHLBI TOPMed Program. *Nature*. 2021;590(7845):290-299. doi:10.1038/S41586-021-03205-Y

4.	Ani A, van der Most PJ, Snieder H, Vaez A, Nolte IM. GWASinspector: comprehensive quality control of genome-wide association study results. *Bioinformatics*. 2021;37(1):129-130. doi:10.1093/bioinformatics/btaa1084

5.	Willer CJ, Li Y, Abecasis GR. METAL: Fast and efficient meta-analysis of genomewide association scans. *Bioinformatics*. 2010;26(17):2190-2191. doi:10.1093/bioinformatics/btq340

6.	Bulik-Sullivan B, Loh PR, Finucane HK, et al. LD score regression distinguishes confounding from polygenicity in genome-wide association studies. *Nat Genet*. 2015;47(3):291-295. doi:10.1038/ng.3211

7.	Yang J, Ferreira T, Morris AP, et al. Conditional and joint multiple-SNP analysis of GWAS summary statistics identifies additional variants influencing complex traits. *Nat Genet*. 2012;44(4):369-375. doi:10.1038/NG.2213

8.	Keaton JM, Kamali Z, Xie T, et al. Genome-wide analysis in over 1 million individuals of European ancestry yields improved polygenic risk scores for blood pressure traits. *Nat Genet*. March 2024. doi:10.21203/rs.3.rs-1409164

9.	Coombes BJ, Ploner A, Bergen SE, Biernacka JM. A principal component approach to improve association testing with polygenic risk scores. *Genet Epidemiol*. 2020;44(7):676-686. doi:10.1002/GEPI.22339

10. Grotzinger AD, Rhemtulla M, de Vlaming R, et al. Genomic structural equation modelling provides insights into the multivariate genetic architecture of complex traits. Nat Hum Behav. 2019;3(5):513-525. doi:10.1038/s41562-019-0566-x

11. Turley P, Walters RK, Maghzian O, et al. Multi-trait analysis of genome-wide association summary statistics using MTAG. Nat Genet. 2018;50(2):229-237. doi:10.1038/s41588-017-0009-4

**Acknowledgements**

**ALSPAC**: We are extremely grateful to all the families who took part in this study, the midwives for their help in recruiting them, and the whole ALSPAC team, which includes interviewers, computer and laboratory technicians, clerical workers, research scientists, volunteers, managers, receptionists and nurses. **Gecko Drenthe**: We are grateful to the families who took part in the GECKO Drenthe study, the midwives, gyneacologists, nurses, and the general practitioners and all health professionals at the Preventive Child Healthcare Drenthe for their help in the recruitment and the measurements, and the GECKO Drenthe study team. **GENR**: The Generation R Study is conducted by Erasmus MC in close collaboration with the School of Law and Faculty of Social Sciences of Erasmus University Rotterdam, the Municipal Health Service Rotterdam area, Rotterdam, the Rotterdam Homecare Foundation, Rotterdam and the Stichting Trombosedienst & Artsenlaboratorium Rijnmond (STAR-MDC), Rotterdam. We gratefully acknowledge the contribution of children and parents, general practitioners, hospitals, midwives and pharmacies in Rotterdam. The generation and management of GWAS genotype data for the Generation R Study were done at the Genetic Laboratory of the Department of Internal Medicine, Erasmus MC, The Netherlands. We would like to thank Karol Estrada, Dr. Tobias A. Knoch, Anis Abuseiris, Luc V. de Zeeuw, and Rob de Graaf, for their help in creating GRIMP, BigGRID, MediGRID, and Services@MediGRID/D-Grid, (funded by the German Bundesministerium fuer Forschung und Technology; grants 01 AK 803 A-H, 01 IG 07015 G) for access to their grid computing resources. We thank Mila Jhamai, Manoushka Ganesh, Pascal Arp, Marijn Verkerk, Lizbeth Herrera and Marjolein Peters for their help in creating, managing and QC of the GWAS database. Also, we thank Karol Estrada for his support in creation and analysis of imputed data. **GINIplus&LISA**: The authors thank all families for participation in the studies and the LISA and GINIplus study teams for their excellent work. **Glaku**: We thank all the GLAKU children and their parents for their enthusiastic participation. We also thank all the research nurses, research assistants, and laboratory personnel involved in the GLAKU study. **HELIX**: We would like to thank all the children and their families for their generous contribution. **INMA and INMA-GIP**: The authors would like to thank all the participants for their generous collaboration. A full roster of the INMA Project Investigators can be found at <http://www.proyectoinma.org/presentacion-inma/listado-investigadores/en_listado-investigadores.html>. **KOALA**: We thank the children and parents from the KOALA study for their participation. The blood pressure monitors were kindly provided by Omron Healthcare Europe BV, Hoofddorp, the Netherlands. **Lifelines**: We are grateful to everyone who participated in the cohort or worked on the cohort to make it possible. **NFBC1986**: We thank all cohort members and researchers who have participated in the study. We also wish acknowledge the work of the NFBC project center. **Project Viva**: We would like to thank the staff and participants of Project Viva. **TDCOB-cases and TDCOB-controls**: The study is part of The Danish Childhood Obesity Data- and Biobank (a.k.a. The HOLBAEK Study); ClinicalTrials.gov ID-no.: NCT00928473. **TRAILS-cc and TRAILS-pop**: We are grateful to everyone who participated in TRAILS or worked on this project to make it possible. ABCD: We thank all participating hospitals, obstetric clinics, general practitioners and primary schools for their assistance in implementing the ABCD study (Amsterdam Born Children and their Development). We also gratefully acknowledge all the women and children who participated in this study for their cooperation.

We thank the ICBP consortium for providing summary statistics.

**UMCG Genetics Lifelines Initiative (UGLI) group author LifeLines Cohort Study:**

Raul Aguirre-Gamboa (1), Patrick Deelen (1), Lude Franke (1), Jan A Kuivenhoven (2), Esteban A Lopera Maya (1), Ilja M Nolte (3), Serena Sanna (1), Harold Snieder (3), Morris A Swertz (1), Peter M. Visscher (3,4), Judith M Vonk (3), Cisca Wijmenga (1)

(1) Department of Genetics, University of Groningen, University Medical Center

Groningen, The Netherlands

(2) Department of Pediatrics, University of Groningen, University Medical Center

Groningen, The Netherlands

(3) Department of Epidemiology, University of Groningen, University Medical Center

Groningen, The Netherlands

(4) Institute for Molecular Bioscience, The University of Queensland, Brisbane,

Queensland, Australia

Authors & Contributors from the International Consortium of Blood Pressure (ICBP) associated with the 2018 Nature Genetics publication: “Genetic analysis of over one million people identifies 535 new loci associated with blood pressure traits.”

Evangelos Evangelou^1,2^, Helen R Warren^3,4^, He Gao^1,5^, Georgios Ntritsos^2^, Niki Dimou^2^, Tonu Esko^16,17^, Reedik Mägi^16^, Lili Milani^16^, Peter Almgren^18^, Thibaud Boutin^19^, Stéphanie Debette^20,21^, Jun Ding^22^, Franco Giulianini^23^, Elizabeth G Holliday^24^, Anne U Jackson^25^, Ruifang Li-Gao^26^, Wei-Yu Lin^27^, Jian'an Luan^28^, Massimo Mangino^29,30^, Christopher Oldmeadow^24^, Bram Peter Prins^31^, Yong Qian^22^, Muralidharan Sargurupremraj^21^, Nabi Shah^32,33^, Praveen Surendran^27^, Sébastien Thériault^34,35^, Niek Verweij^17,36,37^, Sara M Willems^28^, Jing-Hua Zhao^28^, Philippe Amouyel^38^, John Connell^39^, Renée de Mutsert^26^, Alex SF Doney^32^, Martin Farrall^40,41^, Cristina Menni^29^, Andrew D Morris^42^, Raymond Noordam^43^, Guillaume Paré^34^, Neil R Poulter^44^, Denis C Shields^45^, Alice Stanton^46^, Simon Thom^47^, Gonçalo Abecasis^48^, Najaf Amin^49^, Dan E Arking^50^, Kristin L Ayers^51,52^, Caterina M Barbieri^53^, Chiara Batini^54^, Joshua C Bis^55^, Tineka Blake^54^, Murielle Bochud^56^, Michael Boehnke^25^, Eric Boerwinkle^57^, Dorret I Boomsma^58^, Erwin P Bottinger^59^, Peter S Braund^60,61^, Marco Brumat^62^, Archie Campbell^63,64^, Harry Campbell^65^, Aravinda Chakravarti^50^, John C Chambers^1,5,66-68^, Ganesh Chauhan^69^, Marina Ciullo^70,71^, Massimiliano Cocca^72^, Francis Collins^73^, Heather J Cordell^51^, Gail Davies^74,75^, Martin H de Borst^76^, Eco J de Geus^58^, Ian J Deary^74,75^, Joris Deelen^77^, Fabiola Del Greco M^78^, Cumhur Yusuf Demirkale^79^, Marcus Dörr^80,81^, Georg B Ehret^50,82^, Roberto Elosua^83,84^, Stefan Enroth^85^, A Mesut Erzurumluoglu^54^, Teresa Ferreira^86,87^, Mattias Frånberg^88-90^, Oscar H Franco^91^, Ilaria Gandin^62^, Paolo Gasparini^62,72^, Vilmantas Giedraitis^92^, Christian Gieger^93-95^, Giorgia Girotto^62,72^, Anuj Goel^40,41^, Alan J Gow^74,96^, Vilmundur Gudnason^97,98^, Xiuqing Guo^99^, Ulf Gyllensten^85^, Anders Hamsten^88,89^, Tamara B Harris^100^, Sarah E Harris^63,74^, Catharina A Hartman^101^, Aki S Havulinna^102,103^, Andrew A Hicks^78^, Edith Hofer^104,105^, Albert Hofman^91,106^, Jouke-Jan Hottenga^58^, Jennifer E Huffman^19,107,108^, Shih-Jen Hwang^107,108^, Erik Ingelsson^109,110^, Alan James^111,112^, Rick Jansen^113^, Marjo-Riitta Jarvelin^1,5,114-116^, Roby Joehanes^107,117^, Åsa Johansson^85^, Andrew D Johnson^107,118^, Peter K Joshi^65^, Pekka Jousilahti^102^, J Wouter Jukema^119^, Antti Jula^102^, Mika Kähönen^120,121^, Sekar Kathiresan^17,36,122^, Bernard D Keavney^123,124^, Kay-Tee Khaw^125^, Paul Knekt^102^, Joanne Knight^126^, Ivana Kolcic^127^, Jaspal S Kooner^5,67,68,128^, Seppo Koskinen^102^, Kati Kristiansson^102^, Zoltan Kutalik^56,129^, Maris Laan^130^, Marty Larson^107^, Lenore J Launer^100^, Benjamin Lehne^1^, Terho Lehtimäki^131,132^, David CM Liewald^74,75^, Li Lin^82^, Lars Lind^133^, Cecilia M Lindgren^40,87,134^, YongMei Liu^135^, Ruth JF Loos^28,59,136^, Lorna M Lopez^74,137,138^, Yingchang Lu^59^, Leo-Pekka Lyytikäinen^131,132^, Anubha Mahajan^40^, Chrysovalanto Mamasoula^139^, Jaume Marrugat^83^, Jonathan Marten^19^, Yuri Milaneschi^140^, Anna Morgan^62^, Andrew P Morris^40,141^, Alanna C Morrison^142^, Peter J Munson^79^, Mike A Nalls^143,144^, Priyanka Nandakumar^50^, Christopher P Nelson^60,61^, Teemu Niiranen^102,145^, Ilja M Nolte^146^, Teresa Nutile^70^, Albertine J Oldehinkel^147^, Ben A Oostra^49^, Paul F O'Reilly^148^, Elin Org^16^, Sandosh Padmanabhan^64,149^, Walter Palmas^150^, Aarno Palotie^103,151,152^, Alison Pattie^75^, Brenda WJH Penninx^140^, Markus Perola^102,103,153^, Annette Peters^94,95,154^, Ozren Polasek^127,155^, Peter P Pramstaller^78,156,157^, Quang Tri Nguyen^79^, Olli T Raitakari^158,159^, Rainer Rettig^161^, Kenneth Rice^162^, Paul M Ridker^23,163^, Janina S Ried^94^, Harriëtte Riese^147^, Samuli Ripatti^103,164^, Antonietta Robino^72^, Lynda M Rose^23^, Jerome I Rotter^99^, Igor Rudan^165^, Daniela Ruggiero^70,71^, Yasaman Saba^166^, Cinzia F Sala^53^, Veikko Salomaa^102^, Nilesh J Samani^60,61^, Antti-Pekka Sarin^103^, Reinhold Schmidt^104^, Helena Schmidt^166^, Nick Shrine^54^, David Siscovick^167^, Albert V Smith^97,98^, Harold Snieder^146^, Siim Sõber^130^, Rossella Sorice^70^, John M Starr^74,168^, David J Stott^169^, David P Strachan^170^, Rona J Strawbridge^88,89^, Johan Sundström^133^, Morris A Swertz^171^, Kent D Taylor^99^, Alexander Teumer^81,172^, Martin D Tobin^54^, Maciej Tomaszewski^123,124^, Daniela Toniolo^53^, Michela Traglia^53^, Stella Trompet^119,173^, Jaakko Tuomilehto^174-177^, Christophe Tzourio^21^, André G Uitterlinden^91,178^, Ahmad Vaez^146,179^, Peter J van der Most^146^, Cornelia M van Duijn^49^, Germaine C Verwoert^91^, Veronique Vitart^19^, Uwe Völker^81,180^, Peter Vollenweider^181^, Dragana Vuckovic^62,182^, Hugh Watkins^40,41^, Sarah H Wild^183^, Gonneke Willemsen^58^, James F Wilson^19,65^, Alan F Wright^19^, Jie Yao^99^, Tatijana Zemunik^184^, Weihua Zhang^1,67^, John R Attia^24^, Adam S Butterworth^27,185^, Daniel I Chasman^23,163^, David Conen^186,187^, Francesco Cucca^188,189^, John Danesh^27,185^, Caroline Hayward^19^, Joanna MM Howson^27^, Markku Laakso^190^, Edward G Lakatta^191^, Claudia Langenberg^28^, Olle Melander^18^, Dennis O Mook-Kanamori^26,192^, Colin NA Palmer^32^, Lorenz Risch^193-195^, Robert A Scott^28^, Rodney J Scott^24^, Peter Sever^128^, Tim D Spector^29^, Pim van der Harst^196^, Nicholas J Wareham^28^, Eleftheria Zeggini^31^, Daniel Levy^107,118^, Patricia B Munroe^3,4^, Christopher Newton-Cheh^134,197,198^, Morris J Brown^3,4^, Andres Metspalu^16^, Bruce M. Psaty^201,202^, Louise V Wain^54^, Paul Elliott^1,5,203-205^, Mark J Caulfield^3,4^

1.	Department of Epidemiology and Biostatistics, Imperial College London, London, UK.

2.	Department of Hygiene and Epidemiology, University of Ioannina Medical School, Ioannina, Greece.

3.	William Harvey Research Institute, Barts and The London School of Medicine and Dentistry, Queen Mary University of London, London, UK.

4.	National Institute for Health Research, Barts Cardiovascular Biomedical Research Center, Queen Mary University of London, London, UK.

5.	MRC-PHE Centre for Environment and Health, Imperial College London, London, UK.

7.	Division of Epidemiology, Department of Medicine, Institute for Medicine and Public Health, Vanderbilt Genetics Institute, Vanderbilt University Medical Center, Tennessee Valley Healthcare System (626)/Vanderbilt University, Nashville, TN, USA.

8.	Vanderbilt Genetics Institute, Vanderbilt Epidemiology Center, Department of Obstetrics and Gynecology, Vanderbilt University Medical Center; Tennessee Valley Health Systems VA, Nashville, TN, USA.

9.	Department of Epidemiology, Emory University Rollins School of Public Health, Atlanta, GA, USA.

10.	Department of Biomedical Informatics, Emory University School of Medicine, Atlanta, GA, USA.

11.	Massachusetts Veterans Epidemiology Research and Information Center (MAVERIC), VA Boston Healthcare System, Boston, USA.

12.	Division of Aging, Department of Medicine, Brigham and Women’s Hospital, Boston, MA, Department of Medicine, Harvard Medical School, Boston, MA, USA.

13.	Atlanta VAMC and Emory Clinical Cardiovascular Research Institute, Atlanta, GA, USA.

14.	VA Palo Alto Health Care System; Division of Cardiovascular Medicine, Stanford University School of Medicine, CA, USA.

15.	Nephrology Section, Memphis VA Medical Center and University of Tennessee Health Science Center, Memphis, TN, USA.

16.	Estonian Genome Center, University of Tartu, Tartu, Estonia.

17.	Program in Medical and Population Genetics, Broad Institute of Harvard and MIT, Cambridge, MA, USA.

18.	Department Clinical Sciences, Malmö, Lund University, Malmö, Sweden.

19.	MRC Human Genetics Unit, MRC Institute of Genetics and Molecular Medicine, University of Edinburgh, Western General Hospital, Edinburgh, Scotland, UK

20.	Department of Neurology, Bordeaux University Hospital, Bordeaux, France.

21.	Univ. Bordeaux, Inserm, Bordeaux Population Health Research Center, CHU Bordeaux, Bordeaux, France.

22.	Laboratory of Genetics and Genomics, NIA/NIH , Baltimore, MD, USA.

23.	Division of Preventive Medicine, Brigham and Women's Hospital, Boston, MA, USA.

24.	Hunter Medical Reseach Institute and Faculty of Health, University of Newcastle, New Lambton Heights, New South Wales, Australia.

25.	Department of Biostatistics and Center for Statistical Genetics, University of Michigan, Ann Arbor, MI, USA.

26.	Department of Clinical Epidemiology, Leiden University Medical Center, Leiden, the Netherlands.

27.	MRC/BHF Cardiovascular Epidemiology Unit, Department of Public Health and Primary Care, University of Cambridge, Cambridge, UK.

28.	MRC Epidemiology Unit, University of Cambridge School of Clinical Medicine, Cambridge, UK.

29.	Department of Twin Research and Genetic Epidemiology, Kings College London, London, UK.

30.	NIHR Biomedical Research Centre at Guy’s and St Thomas’ Foundation Trust, London, UK.

31.	Wellcome Trust Sanger Institute, Hinxton, UK.

32.	Division of Molecular and Clinical Medicine, School of Medicine, University of Dundee, UK.

33.	Department of Pharmacy, COMSATS Institute of Information Technology, Abbottabad, Pakistan.

34.	Department of Pathology and Molecular Medicine, McMaster University, Hamilton, Canada.

35.	Institut universitaire de cardiologie et de pneumologie de Québec-Université Laval, , Quebec City, Canada.

36.	Cardiovascular Research Center and Center for Human Genetic Research, Massachusetts General Hospital, Boston, Massachusetts, ΜΑ, USA.

37.	University of Groningen, University Medical Center Groningen, Department of Cardiology, Groningen, The Netherlands.

38.	University of Lille, Inserm, Centre Hosp. Univ Lille, Institut Pasteur de Lille, UMR1167 - RID-AGE - Risk factors and molecular determinants of aging-related diseases, Epidemiology and Public Health Department, Lille, France.

39.	University of Dundee, Ninewells Hospital & Medical School, Dundee, , UK.

40.	Wellcome Trust Centre for Human Genetics, University of Oxford, Oxford, UK.

41.	Division of Cardiovascular Medicine, Radcliffe Department of Medicine, University of Oxford, Oxford, UK.

42.	Usher Institute of Population Health Sciences and Informatics, University of Edinburgh, UK.

43.	Department of Internal Medicine, Section Gerontology and Geriatrics, Leiden University Medical Center, Leiden, The Netherlands.

44.	Imperial Clinical Trials Unit, Stadium House, 68 Wood Lane, London, UK.

45.	School of Medicine, University College Dublin, Ireland.

46.	Molecular and Cellular Therapeutics, Royal College of Surgeons in Ireland, Dublin, Ireland.

47.	International Centre for Circulatory Health, Imperial College London, London, UK.

48.	Center for Statistical Genetics, Dept. of Biostatistics, SPH II, Washington Heights, Ann Arbor, MI, USA.

49.	Genetic Epidemiology Unit, Department of Epidemiology, Erasmus MC, Rotterdam, the Netherlands.

50.	Center for Complex Disease Genomics, McKusick-Nathans Institute of Genetic Medicine, Johns Hopkins University School of Medicine, Baltimore, MD, USA.

51.	Institute of Genetic Medicine, Newcastle University, Newcastle upon Tyne, UK.

52.	Sema4, a Mount Sinai venture, Stamford, CT, USA.

53.	Division of Genetics and Cell Biology, San Raffaele Scientific Institute, Milano, Italy.

54.	Department of Health Sciences, University of Leicester, Leicester, UK.

55.	Cardiovascular Health Research Unit, Department of Medicine, University of Washington, Seattle, WA, USA.

56.	Institute of Social and Preventive Medicine, University Hospital of Lausanne, Lausanne, Switzerland.

57.	Human Genetics Center, School of Public Health, The University of Texas Health Science Center at Houston and Human Genome Sequencing Center, Baylor College of Medicine, One Baylor Plaza, Houston, TX, USA.

58.	Department of Biological Psychology, Vrije Universiteit Amsterdam, EMGO+ institute, VU University medical center, Amsterdam, the Netherlands.

59.	The Charles Bronfman Institute for Personalized Medicine, Icachn School of Medicine at Mount Sinai, NY, USA.

60.	Department of Cardiovascular Sciences, University of Leicester, Leicester, UK.

61.	 NIHR Leicester Biomedical Research Centre, Glenfield Hospital, Groby Road, Leicester, UK.

62.	Department of Medical, Surgical and Health Sciences, University of Trieste, , Trieste, Italy.

63.	Medical Genetics Section, Centre for Genomic and Experimental Medicine, Institute of Genetics and Molecular Medicine, University of Edinburgh, Edinburgh, UK.

64.	Generation Scotland, Centre for Genomic and Experimental Medicine, University of Edinburgh, Edinburgh, UK.

65.	Centre for Global Health Research, Usher Institute of Population Health Sciences and Informatics, University of Edinburgh, Edinburgh, Scotland, UK

66.	Lee Kong Chian School of Medicine, Nanyang Technological University, Singapore, Singapore.

67.	Department of Cardiology, Ealing Hospital, Middlesex, UK.

68.	Imperial College Healthcare NHS Trust, London, UK.

69.	Centre for Brain Research, Indian Institute of Science, Bangalore, India.

70.	Institute of Genetics and Biophysics "A. Buzzati-Traverso", CNR, Napoli, Italy.

71.	IRCCS Neuromed, Pozzilli, Isernia, Italy.

72.	Institute for Maternal and Child Health IRCCS Burlo Garofolo, Trieste, Italy.

73.	Medical Genomics and Metabolic Genetics Branch, National Human Genome Research Institute, NIH, Bethesda, MD, USA.

74.	Centre for Cognitive Ageing and Cognitive Epidemiology, University of Edinburgh, 7 George Square, Edinburgh, UK.

75.	Department of Psychology, University of Edinburgh, 7 George Square, Edinburgh, UK.

76.	Department of Internal Medicine, Division of Nephrology, University of Groningen, University Medical Center Groningen, Groningen, The Netherlands.

77.	Department of Molecular Epidemiology, Leiden University Medical Center, Leiden, the Netherlands.

78.	Institute for Biomedicine, Eurac Research, Bolzano, Italy - Affiliated Institute of the University of Lübeck, Lübeck, Germany.

79.	Mathematical and Statistical Computing Laboratory, Office of Intramural Research, Center for Information Technology, National Institutes of Health, Bethesda, MD, USA.

80.	Department of Internal Medicine B, University Medicine Greifswald, Greifswald, Germany.

81.	DZHK (German Centre for Cardiovascular Research), partner site Greifswald, Greifswald, Germany.

82.	Cardiology, Department of Medicine, Geneva University Hospital, Geneva, Switzerland.

83.	CIBERCV & Cardiovascular Epidemiology and Genetics, IMIM. Dr Aiguader 88, Barcelona, Spain.

84.	Faculty of Medicine, Universitat de Vic-Central de Catalunya, Vic, Spain.

85.	Department of Immunology, Genetics and Pathology, Uppsala Universitet, Science for Life Laboratory, Uppsala, Sweden.

86.	Wellcome Centre for Human Genetics, University of Oxford, Roosevelt Drive, Oxford, UK.

87.	Big Data Institute, Li Ka Shing Center for Health for Health Information and Discovery, Oxford University, Old Road, Oxford, UK.

88.	Cardiovascular Medicine Unit, Department of Medicine Solna, Karolinska Institutet, Stockholm, Sweden.

89.	Centre for Molecular Medicine, L8:03, Karolinska Universitetsjukhuset, Solna, Sweden.

90.	Department of Numerical Analysis and Computer Science, Stockholm University, Stockholm, Sweden.

91.	Department of Epidemiology, Erasmus MC, Rotterdam, the Netherlands.

92.	Department of Public Health and Caring Sciences, Geriatrics, Uppsala, Sweden.

93.	Research Unit of Molecular Epidemiology, Helmholtz Zentrum München, German Research Center for Environmental Health, Neuherberg, Germany.

94.	Institute of Epidemiology, Helmholtz Zentrum München, German Research Center for Environmental Health, Neuherberg, Germany.

95.	German Center for Diabetes Research (DZD e.V.), Neuherberg, Germany.

96.	Department of Psychology, School of Social Sciences, Heriot-Watt University, Edinburgh, UK.

97.	Faculty of Medicine, University of Iceland, Reykjavik, Iceland.

98.	Icelandic Heart Association, Kopavogur, Iceland.

99.	The Institute for Translational Genomics and Population Sciences, Department of Pediatrics, LABioMed at Harbor-UCLA Medical Center, Torrance, CA, USA.

100.	Intramural Research Program, Laboratory of Epidemiology, Demography, and Biometry, National Institute on Aging, Bethesda, MD, USA.

101.	Department of Psychiatry, University of Groningen, University Medical Center Groningen, Groningen, The Netherlands.

102.	Department of Public Health Solutions, National Institute for Health and Welfare (THL), Helsinki, Finland.

103.	Institute for Molecular Medicine Finland (FIMM), University of Helsinki, Helsinki, Finland.

104.	Clinical Division of Neurogeriatrics, Department of Neurology, Medical University of Graz, Graz, Austria.

105.	Institute for Medical Informatics, Statistics and Documentation, Medical University of Graz, Graz, Austria.

106.	Department of Epidemiology, Harvard T.H. Chan School of Public Health, Boston, MA, USA.

107.	National Heart, Lung and Blood Institute's Framingham Heart Study, Framingham, MA, USA.

108.	The Population Science Branch, Division of Intramural Research, National Heart Lung and Blood Institute national Institute of Health, Bethesda, MD, USA.

109.	Department of Medical Sciences, Molecular Epidemiology and Science for Life Laboratory, Uppsala University, Uppsala, Sweden.

110.	Division of Cardiovascular Medicine, Department of Medicine, Stanford University School of Medicine, Stanford, CA USA.

111.	Department of Pulmonary Physiology and Sleep, Sir Charles Gairdner Hospital, Hospital Avenue, Nedlands, Australia.

112.	School of Medicine and Pharmacology, University of Western Australia.

113.	Department of Psychiatry, VU University Medical Center, Amsterdam Neuroscience, Amsterdam, the Netherlands.

114.	Biocenter Oulu, University of Oulu, Oulu, Finland.

115.	Center For Life-course Health Research, University of Oulu, Oulu Finland.

116.	Unit of Primary Care, Oulu University Hospital, Oulu, Oulu, Finland.

117.	Hebrew SeniorLife, Harvard Medical School, Boston, MA, USA.

118.	Population Sciences Branch, National Heart, Lung and Blood Institute, National Institutes of Health, Bethesda, MD, USA.

119.	Department of Cardiology, Leiden University Medical Center, Leiden, the Netherlands.

120.	Department of Clinical Physiology, Tampere University Hospital, Tampere, Finland.

121.	Department of Clinical Physiology, Finnish Cardiovascular Research Center - Tampere, Faculty of Medicine and Life Sciences, University of Tampere, Tampere, Finland.

122.	Broad Institute of the Massachusetts Institute of Technology and Harvard University, Cambridge, MA, USA.

123.	Division of Cardiovascular Sciences, Faculty of Biology, Medicine and Health, The University of Manchester, Manchester, UK.

124.	Division of Medicine, Manchester University NHS Foundation Trust, Manchester Academic Health Science Centre, Manchester, UK

125.	Department of Public Health and Primary Care, Institute of Public Health, University of Cambridge, Cambridge, UK.

126.	Data Science Institute and Lancaster Medical School, Lancaster, UK.

127.	Department of Public Health, Faculty of Medicine, University of Split, Croatia.

128.	National Heart and Lung Institute, Imperial College London, London, UK.

129.	Swiss Institute of Bioinformatics, Lausanne, Switzerland.

130.	Institute of Biomedicine and Translational Medicine, University of Tartu, Tartu, Estonia.

131.	Department of Clinical Chemistry, Fimlab Laboratories, Tampere, Finland.

132.	Department of Clinical Chemistry, Finnish Cardiovascular Research Center - Tampere, Faculty of Medicine and Life Sciences, University of Tampere, Tampere, Finland

133.	Department of Medical Sciences, Cardiovascular Epidemiology, Uppsala University, Uppsala, Sweden.

134.	Program in Medical and Population Genetics, Broad Institute, Cambridge, MA, USA.

135.	Division of Public Health Sciences, Wake Forest School of Medicine, Winston-Salem, NC, USA.

136.	Mindich Child health Development Institute, The Icahn School of Medicine at Mount Sinai, New York, NY, USA.

137.	Department of Psychiatry, Royal College of Surgeons in Ireland, Education and Research Centre, Beaumont Hospital, Dublin, Ireland.

138.	University College Dublin, UCD Conway Institute, Centre for Proteome Research, UCD, Belfield, Dublin, Ireland.

139.	Institute of Health and Society, Newcastle University, Newcastle upon Tyne, UK.

140.	Department of Psychiatry, Amsterdam Public Health and Amsterdam Neuroscience, VU University Medical Center/GGZ inGeest, Amsterdam, The Netherlands.

141.	Department of Biostatistics, University of Liverpool, Block F, Waterhouse Building, Liverpool, UK.

142.	Department of Epidemiology, Human Genetics and Environmental Sciences, School of Public Health, University of Texas Health Science Center at Houston, Houston, TX, USA.

143.	Data Tecnica International, Glen Echo, MD, USA.

144.	Laboratory of Neurogenetics, National Institute on Aging, Bethesda, USA.

145.	Department of Medicine, Turku University Hospital and University of Turku, Finland.

146.	Department of Epidemiology, University of Groningen, University Medical Center Groningen, Groningen, The Netherlands.

147.	Interdisciplinary Center Psychopathology and Emotion regulation (ICPE), University of Groningen, University Medical Center Groningen, Groningen, The Netherlands.

148.	SGDP Centre, Institute of Psychiatry, Psychology and Neuroscience, King's College London, London, UK.

149.	British Heart Foundation Glasgow Cardiovascular Research Centre, Institute of Cardiovascular and Medical Sciences, College of Medical, Veterinary and Life Sciences, University of Glasgow, Glasgow, UK.

150.	Department of Medicine, Columbia University Medical Center, New York, NY, USA.

151.	Analytic and Translational Genetics Unit, Department of Medicine, Department of Neurology and Department of Psychiatry Massachusetts General Hospital, Boston, MA, USA.

152.	The Stanley Center for Psychiatric Research and Program in Medical and Population Genetics, The Broad Institute of MIT and Harvard, Cambridge, MA, USA.

153.	University of Tartu, Tartu, Estonia.

154.	German Center for Cardiovascular Disease Research (DZHK), partner site Munich, Neuherberg, Germany.

155.	Psychiatric hospital “Sveti Ivan”, Zagreb, Croatia.

156.	Department of Neurology, General Central Hospital, Bolzano, Italy.

157.	Department of Neurology, University of Lübeck, Lübeck, Germany.

158.	Department of Clinical Physiology and Nuclear Medicine, Turku University Hospital, Turku, Finland.

159.	Research Centre of Applied and Preventive Cardiovascular Medicine, University of Turku, Turku, Finland.

161.	Institute of Physiology, University Medicine Greifswald, Karlsburg, Germany.

162.	Department of Biostatistics University of Washington, Seattle, WA, USA.

163.	Harvard Medical School, Boston MA.

164.	Public health, Faculty of Medicine, University of Helsinki, Finland

165.	Centre for Global Health Research, Usher Institute of Population Health Sciences and Informatics, University of Edinburgh, Scotland, UK.

166.	Gottfried Schatz Research Center for Cell Signaling, Metabolism & Aging, Molecular Biology and Biochemistry, Medical University of Graz, Graz, Austria.

167.	The New York Academy of Medicine, New York, NY, USA.

168.	Alzheimer Scotland Dementia Research Centre, University of Edinburgh, Edinburgh, UK.

169.	Institute of Cardiovascular and Medical Sciences, Faculty of Medicine, University of Glasgow, United Kingdom.

170.	Population Health Research Institute, St George's, University of London, London, UK.

171.	Department of Genetics, University of Groningen, University Medical Center Groningen, Groningen, The Netherlands.

172.	Institute for Community Medicine, University Medicine Greifswald, Greifswald, Germany.

173.	Department of Gerontology and Geriatrics, Leiden University Medical Center, Leiden, the Netherlands.

174.	Dasman Diabetes Institute, Dasman, Kuwait.

175.	Chronic Disease Prevention Unit, National Institute for Health and Welfare, Helsinki, Finland.

176.	Department of Public Health, University of Helsinki, Helsinki, Finland.

177.	Saudi Diabetes Research Group, King Abdulaziz University, Jeddah, Saudi Arabia.

178.	Department of Internal Medicine, Erasmus MC, Rotterdam, the Netherlands.

179.	Research Institute for Primordial Prevention of Non-communicable Disease, Isfahan University of Medical Sciences, Isfahan, Iran.

180.	Interfaculty Institute for Genetics and Functional Genomics, University Medicine Greifswald, Greifswald, Germany.

181.	Department of Internal Medicine, University Hospital, CHUV, Lausanne, Switzerland.

182.	Experimental Genetics Division, Sidra Medical and Research Center, Doha, Qatar.

183.	Centre for Population Health Sciences, Usher Institute of Population Health Sciences and Informatics, University of Edinburgh, Scotland, UK

184.	Department of Biology, Faculty of Medicine, University of Split, Croatia.

185.	The National Institute for Health Research Blood and Transplant Research Unit in Donor Health and Genomics, University of Cambridge, UK.

186.	Division of Cardiology, University Hospital, Basel, Switzerland.

187.	Division of Cardiology, Department of Medicine, McMaster University, Hamilton, Canada.

188.	Institute of Genetic and Biomedical Research, National Research Council (CNR), Monserrato, Cagliari, Italy.

189.	Department of Biomedical Sciences, University of Sassari, Sassari, Italy.

190.	Institute of Clinical Medicine, Internal Medicine, University of Eastern Finland and Kuopio University Hospital, Kuopio, Finland.

191.	Laboratory of Cardiovascular Science, NIA/NIH , Baltimore, MD, USA.

192.	Department of Public Health and Primary Care, Leiden University Medical Center, Leiden, the Netherlands.

193.	Labormedizinisches Zentrum Dr. Risch, Schaan, Liechtenstein.

194.	Private University of the Principality of Liechtenstin, Triesen, Liechtenstein.

195.	University Insitute of Clinical Chemistry, Inselspital, Bern University Hospital, University of Bern, Bern, Switzerland.

196.	Department of Cardiology, University of Groningen, University Medical Center Groningen, Groningen, The Netherlands.

197.	Center for Genomic Medicine, Massachusetts General Hospital, Boston, MA, USA.

198.	Cardiovascular Research Center, Massachusetts General Hospital, Boston, MA, USA.

201.	Cardiovascular Health Research Unit, Departments of Medicine, Epidemiology and Health Services, University of Washington, Seattle, WA, USA.

202.	Kaiser Permanente Washington Health Research Institute, Seattle, WA, USA.

203.	National Institute for Health Research Imperial Biomedical Research Centre, Imperial College Healthcare NHS Trust and Imperial College London, London, UK.

204.	UK Dementia Research Institute (UK DRI) at Imperial College London, London, UK

205.	Health Data Research-UK London substantive site, London, U.K

**Sources of Funding**

**ALSPAC**: "The UK Medical Research Council and Wellcome (Grant ref: 217065/Z/19/Z) and the University of Bristol provide core support for ALSPAC. A comprehensive list of grants funding is available on the ALSPAC website (http://www.bristol.ac.uk/alspac/external/documents/grant-acknowledgements.pdf). This research was funded in whole, or in part, by the Wellcome Trust (WT088806). For the purpose of Open Access, the authors have applied a CC BY public copyright licence to any Author Accepted Manuscript version arising from this submission. GWAS data was generated by Sample Logistics and Genotyping Facilities at Wellcome Sanger Institute and LabCorp (Laboratory Corporation of America) using support from 23andMe. ALGS, NJT and DAL work in a Unit that is supported by the University of Bristol and UK Medical Research Council (MC_UU_00011/1 and MC_UU_00011/6), NJT and DAL work in the Bristol NIHR Biomedical Research Centre, and NJT works within the CRUK Integrative Cancer Epidemiology Programme (C18281/A29019). ALGS’ contribution to this research is supported by the study of Dynamic longitudinal exposome trajectories in cardiovascular and metabolic non-communicable diseases (H2020-SC1-2019-Single-Stage-RTD, project ID 874739). D.A.L’s contribution is supported by the UK Medical Research Council (MC_UU_00032/05) and British Heart Foundation ((CH/F/20/90003). NJT is a Wellcome Trust Investigator (202802/Z/16/Z), and he is the PI of the Avon Longitudinal Study of Parents and Children (MRC & WT 217065/Z/19/Z). None of the grant funders or those acknowledged influenced the study design or interpretation of results. The views expressed in this paper are those of the authors and not necessarily those of funders or acknowledged.

**BAMSE**: The Swedish Research Council, the Swedish Heart-Lung Foundation, Strategic Research Area (SFO) Epidemiology, Karolinska Institutet and Region Stockholm (ALF project, and for cohort and database maintenance).

**COPSAC 2000** **and COPSAC 2010**: All funding received by COPSAC is listed on www.copsac.com. The Lundbeck Foundation (Grant no R16-A1694); The Ministry of Health (Grant no 903516); Danish Council for Strategic Research (Grant no 0603-00280B) and The Capital Region Research Foundation have provided core support to the COPSAC research center.

EDEN: EDEN study was funded by Foundation for Medical Research (FRM), National Agency for Research (ANR), National Institute for Research in Public Health (IRESP: TGIR cohorte sante 2008 program), French Ministry of Health (DGS), French Ministry of Research, Inserm Bone and Joint Diseases National Research (PRO-A) and Human Nutrition National Research Programs, Paris–Sud University, Nestle´, French National Institute for Population Health Surveillance (InVS), French National Institute for Health Education (INPES), the European Union FP7 programmes (FP7/2007-2013, HELIX, ESCAPE, ENRIECO, Medall projects), Diabetes National Research Program (through a collaboration with the French Association of Diabetic Patients (AFD)), French Agency for Environmental Health Safety (now ANSES), Mutuelle Generale de l’Education Nationale (MGEN), French National Agency for Food Security, and the French-speaking association for the study of diabetes and metabolism (ALFEDIAM).

**Gecko Drenthe**: The GECKO Drenthe birth cohort was funded by an unrestricted grant of Hutchison Whampoa Ld, Hong Kong and supported by the University of Groningen, Well Baby Clinic Foundation Icare, Noordlease, Paediatric Association Of The Netherlands, Youth Preventive Health Care Drenthe and European Union’s Horizon 2020 research and innovation programme (LifeCycle, Grant Agreement No. 733206 LifeCycle).

**GENR:**	The general design of Generation R Study is made possible by financial support from Erasmus MC, Rotterdam, Erasmus University Rotterdam, the Netherlands Organization for Health Research and Development (ZonMw), the Netherlands Organisation for Scientific Research (NWO), the Ministry of Health, Welfare and Sport and the Ministry of Youth and Families. V.W.V.J. received a grant from the European Research Council (ERC-2014-CoG-648916). This project received funding from the European Union’s Horizon 2020 Research and Innovation Programme (733206, LIFECYCLE, 874739, LongITools).

**Glaku**:	The study has been supported by Academy of Finland, University of Helsinki, Hope and Optimism Initiative, Finnish Foundation for Pediatric Research, Sigrid Juselius Foundation, Jalmari and Rauha Ahokas Foundation, Signe and Ane Gyllenberg Foundation, Yrjo Jahnsson Foundation, Juho Vainio Foundation, Emil Aaltonen Foundation, and Ministry of Education and Culture, Finland. The 352 samples were genotyped at the Genotyping and Sequencing Core Facility of the Estonian Genome Centre, University of Tartu.

**GPI**: GPI cohorts (GSH, EpiGo, LACHY) have been funded by NIH HL069999, HL105689, and HL064157.

**HELIX**:	This work was supported by the European Community’s Seventh Framework Programme (FP7/2007-206) [grant agreement no 308333 (HELIX project)]. The genotyping was supported by Instituto de Salud Carlos III [PI17/01225]; and co-funded by European Union (ERDF, “A way to make Europe”) and the Centro Nacional de Genotipado-CEGEN [PRB2-ISCIII]. KANC was funded by the grant of the Lithuanian Agency for Science Innovation and Technology [6-04-2014_31V-66]. The Norwegian Mother, Father and Child Cohort Study is supported by the Norwegian Ministry of Health and Care Services and the Ministry of Education and Research. The Rhea project was financially supported by European projects [EU FP6-2003-Food-3-NewGeneris, EU FP6. STREP Hiwate, EU FP7 ENV.2007.1.2.2.2. Project No 211250 Escape, EU FP7-2008-ENV-1.2.1.4 Envirogenomarkers, EU FP7-HEALTH-2009- single stage CHICOS, EU FP7 ENV.2008.1.2.1.6. Proposal No 226285 ENRIECO, EU- FP7- HEALTH-2012 Proposal No 308333 HELIX], and the Greek Ministry of Health [Program of Prevention of obesity and neurodevelopmental disorders in preschool children, in Heraklion district, Crete, Greece: 2011-2014; “Rhea Plus”: Primary Prevention Program of Environmental Risk Factors for Reproductive Health, and Child Health: 2012-15]. We acknowledge support from the Spanish Ministry of Science and Innovation through the “Centro de Excelencia Severo Ochoa 2019-2023” Program [CEX2018-000806-S], and support from the Generalitat de Catalunya through the CERCA Program.

**INMA**:	This study was funded by grants from Instituto de Salud Carlos III (CB06/02/0041, G03/176, FIS PI041436, PI081151, PI041705, and PS09/00432, FIS-FEDER 03/1615, 04/1509, 04/1112, 04/1931 , 05/1079, 05/1052, 06/1213, 07/0314, and 09/02647), Fundació La Marató de TV3 (090430), Generalitat de Catalunya-CIRIT (1999SGR 00241), Conselleria de Sanitat Generalitat Valenciana, and Fundación Roger Torné. ISGlobal is a member of the CERCA Programme, Generalitat de Catalunya.

**INMA-GIP**: The INMA-GIP cohort was funded by the Spanish Biomedical Research Center in Epidemiology and Public Health (CIBERESP) and by grants from the Instituto de Salud Carlos III (PI06/0867, PI09/00090, PI13/02187 and PI18/01142) cofunded by ERDF, “A way to make Europe”, the Basque Department of Health (Projects 2005111093, 2009111069, 2013111089, 2015111065 and 2018111086), the Provincial Government of Gipuzkoa (Projects DFG06/002, DFG08/001 and DFG15/221) and annual agreements with the municipalities of the study area (Zumarraga, Urretxu, Legazpi, Azkoitia, Azpeitia and Beasain).

**KOALA**: The following funds contributed to the data collection and genotyping: Netherlands Asthma Foundation (grants no. 3.2.07.022 & 3.2.03.48); Stichting Astma Bestrijding; Dutch Biobanking and Biomolecular Resources Infrastructure (BBMRI-NL); Netherlands Organisation for Health Research and Development (ZONmw Enabling Technologies 2013 grant no. 435000021).

**Lifelines**: The Lifelines initiative has been made possible by subsidy from the Dutch Ministry of Health, Welfare and Sport, the Dutch Ministry of Economic Affairs, the University Medical Center Groningen (UMCG), Groningen University and the Provinces in the North of the Netherlands (Drenthe, Friesland, Groningen).

**NFBC1986**: EU QLG1-CT-2000-01643 (EUROBLCS) Grant no. E51560, NorFA Grant no. 731, 20056, 30167, USA / NIH 2000 G DF682 Grant no. 50945

**Project Viva**: Project Viva has been funded by National Institutes of Health (R01 HD 034568). In addition, Project Viva acknowledges the contributions of Dr. Steven Zeisel, whose laboratory provided support for Project Viva genotyping.

**Raine Study**: The Raine Study was supported by the National Health and Medical Research Council of Australia [grant numbers 572613, 403981, 1059711], the Canadian Institutes of Health Research [grant number MOP-82893]. The authors gratefully acknowledge all Raine Study participants and their families for their continued participation in the study, as well as the Raine Study team for study co-ordination and data collection. We also thank the NHMRC and the Raine Medical Research Foundation for their support. The core management of the Raine Study is funded by The University of Western Australia, Curtin University, The Kids Research Institute Australia, Women and Infants Research Foundation, Edith Cowan University, Murdoch University, The University of Notre Dame Australia and the Western Australian Future Health Research and Innovation Fund (2023-2024; Grant ID WACSOSP2023-2024). This work was supported by resources provided by the Pawsey Supercomputing Centre with funding from the Australian Government and Government of Western Australia.

**SFS**: Financial support was provided by Agence Nationale de la Recherche, programme d’Investissements d’avenir, grant number ANR-15RHU-0004.

**TDCOB-cases and TDCOB-controls**: The Innovation Fund Denmark, grant number: 0603-00484B (TARGET); The Novo Nordisk Foundation, grant number: NNF15OC0016544, NNF23SA0084103; The MicrobLiver Challenge Grant (NNF15OC0016692). Further, Cilius Esmann Fonvig was supported by the BRIDGE – Translational Excellence Programme (grant number: NNF18SA0034956), Steno Diabetes Center Sjaelland, and The Region Zealand Health Scientific Research Foundation. Louise Aas Holm was supported by A.P. Møller Fonden and a research grant from the Danish Cardiovascular Academy (PhD2023009-HF), which is funded by the Novo Nordisk Foundation, grant number NNF20SA0067242 and The Danish Heart Foundation.

**TRAILS-cc and TRAILS-pop**: This research is part of the TRacking Adolescents' Individual Lives Survey (TRAILS). Participating centers of TRAILS include various departments of the University Medical Center and University of Groningen, the University of Utrecht, the Radboud Medical Center Nijmegen, and the Parnassia Bavo group, all in the Netherlands. TRAILS has been financially supported by various grants from the Netherlands Organization for Scientific Research (NWO), ZonMW, GB-MaGW, the Dutch Ministry of Justice, the European Science Foundation, the European Research Council, BBMRI-NL, and the participating universities. Statistical analyses were carried out on the Genetic Cluster Computer (http://www.geneticcluster.org), which is financially supported by the Netherlands Scientific Organization (NWO 480-05-003) along with a supplement from the Dutch Brain Foundation.

**TMM BirThree Cohort Study**: Japan Agency for Medical Research and Development (AMED) [grant numbers, JP17km0105001, JP21tm0124005, and JP21tm0424601].

**ABCD:** The ABCD study (Amsterdam Born Children and their Development) has been supported by grants from The Netherlands Organisation for Health Research and Development (ZonMW) and The Netherlands Heart Foundation. Genotyping was funded by the BBMRI-NL grant CP2013-50. T.G.M. Vrijkotte was supported by ZonMW (TOP 40–00812–98–11010).

Tian Xie is supported by the National Natural Science Foundation of China (Grant No. 32500516), the Guangdong Provincial High-level Talent Program (Grant No. 2024QN11Y205), and the Young Scientists Program of Guangzhou Laboratory (Grant No. QNPG24-16).

**Supplementary Figures**

Figure S1. Manhattan plot of genome-wide MAP associations. The x-axis represents the genome in physical order; the y-axis shows −log_10_ P values for individual SNP association with MAP from the meta-analyses in European children (N = 28,425). Genome-wide significance at P value < 5 × 10^−8^ is indicated by the red line. Lead SNPs are annotated with the nearest genes. MAP indicates mean arterial pressure.

Figure S2. Quantile-Quantile plots of the SNPs in the meta-analyses of childhood BP traits and HR. DBP indicates diastolic blood pressure; HR, heart rate; MAP, mean arterial pressure; PP, pulse pressure; SBP, systolic blood pressure. The observed linkage disequilibrium score regression (LDSC) intercepts were 0.9933, 0.9963, 0.995, 0.9955, 1.0105 for SBP, DBP, MAP, PP, and HR respectively, which indicated no inflation.

1) rs113573379 for SBP

2) rs12258967 for SBP

3) rs6060954 for SBP

4) rs11190709 for DBP

5) rs872588 for DBP

6) rs79493618 for MAP

7) rs12258967 for MAP

8) rs11190709 for MAP

9) rs6060954 for MAP

10) rs79958663 for PP

11) rs4984497 for PP

12) rs1011121 for PP

13) rs142556838 for HR

14) rs17884589 for HR

15) rs445754 for HR

Figure S3. Regional plots for lead SNPs of childhood BP traits and HR. Linkage Disequilibrium is calculated using the 1000 Genome European population with grey color corresponding to r^2^< 0.1. DBP indicates diastolic blood pressure; HR, heart rate; MAP, mean arterial pressure; PP, pulse pressure; SBP, systolic blood pressure.

Figure S4. Comparing effect sizes between adults and children of European ethnicity for genome-wide significant SNPs reported by adulthood meta-GWAS of BP traits and HR. DBP indicates diastolic blood pressure; HR, heart rate; MAP, mean arterial pressure; PP, pulse pressure; SBP, systolic blood pressure.

Figure S5. Comparison of absolute adult effect sizes between shared and non-shared loci. Each panel shows Wilcoxon p-value testing whether shared SNPs have larger adult effects. Significance status (0 = not shared; 1 = shared, i.e. SNPs significant in both childhood and adult BP or HR GWAS). Boxes represent the interquartile range (IQR), the horizontal line denotes the median, and outliers beyond whiskers are values below Q1−1.5×IQR or above Q3+1.5×IQR. DBP indicates diastolic blood pressure; HR, heart rate; MAP, mean arterial pressure; PP, pulse pressure; SBP, systolic blood pressure.

**Figure S6. Quintile analyses of child-based and adult-based PRSs in cohorts of three ancestries.** EUR-3 years: European children at age 3 from EDEN cohort (N=574); EUR-5 years: European children at age 5 from EDEN cohort (N=522); EUR-6 years: European children at age 6 from Amsterdam Born Children and their Development (ABCD) cohort (N=1147); EUR-11 years: European children at age 11 from ABCD cohort (N=369); EA1 and EA2: a cohort of East-Asian children (TMM BirThree Cohort Study, divided into two sub cohorts: N= 572 with genotyping chip JPAv2 and N=542 with chip NEO, mean age=10.2 years with age range from 7 to 17 years); AFR: a cohort of African-American children (GPI, N=835, mean age=14.7 years with age range from 4 to 17 years). DBP indicates diastolic blood pressure; HR, heart rate; PP, pulse pressure; SBP, systolic blood pressure. Values represent mean differences relative to the lowest PRS quintile. *P value < 0.05. 95% confidence intervals and exact P values are provided in Table S15b.

Figure S7. Flowchart of the inclusion of study participants in the UK Biobank for the PheWAS. PRS indicates polygenic risk score.

Figure S8. Common factor model in GenomicSEM including DBP, SBP and HR. The edges in the figure show the correlational values (i.e. how close the manifest value resembles the latent factor) and the residual values at the bottom of the manifest values (only HR has significant residuals). Model fit indices: comparative fit index (CFI) = 0.931; standardized root mean square residual (SRMR) = 0.186.
